# Cytoplasmic cleavage of DPPA3 is required for intracellular trafficking and cleavage-stage development in mice

**DOI:** 10.1038/s41467-017-01387-6

**Published:** 2017-11-21

**Authors:** Seung-Wook Shin, Edgar John Vogt, Maria Jimenez-Movilla, Boris Baibakov, Jurrien Dean

**Affiliations:** 1 Laboratory of Cellular and Developmental Biology, NIDDK, National Institutes of Health, Bethesda, MD 20892 USA; 2 Department of Cell Biology and Histology, Medical School, University of Murcia, IMIB, 30100 Murcia, Spain

## Abstract

Degradation of maternal proteins by the ubiquitin-proteasome system (UPS) accompanies the maternal-to-zygotic transition. DPPA3/Stella/PGC7, encoded by a maternal effect gene, is present in the nucleus and cytoplasm of zygotes and has been associated with protecting the female pronucleus from TET3-mediated demethylation. We now report that cytoplasmic DPPA3 is partially cleaved by the ubiquitin-proteasome system and an N-terminus fragment remains in the cytoplasm where it associates with early and re-cycling endosomes. If DPPA3 is absent or if cleavage is prevented, multiple vesicles coalesce/aggregate and markers of lysosomes are decreased. Fertilized eggs develop poorly into blastocysts, which results in significantly decreased fecundity of *Dppa3*^*R60A*^ transgenic mice. This phenocopies aspects of *Lamp1/2* knockdowns and *Dppa3*^*KO*^ embryos can be partially rescued in vitro by DPPA3^1–60^ and to a lesser extent by LAMP1/2. Thus, the N-terminus of DPPA3 has a significant role in cytoplasmic vesicular trafficking in addition to its previously reported nuclear function.

## Abstract (web summary)

During the maternal-to-zygotic transition there is degradation of maternal proteins via the ubiquitin-proteasome system (UPS). Here, the authors show that in the embryo the maternally inherited epigenetic factor DPPA3/Stella/PGC7 is cleavage by the UPS and controls intracellular trafficking.

## Introduction

Maternal effect genes encode proteins that accumulate during oogenesis and are essential for cleavage-stage embryonic development. Many of these same proteins undergo proteolytic processing by the ubiquitin-proteasome system (UPS) in the early embryo. This ostensible paradox is resolved if the proteolytic degradation products serve a biological imperative that results in multiple tasks for the single processed protein. The UPS is highly conserved across species and participates in a variety of biological processes^1–3^. Mostly commonly, the post-translational cleavage is complete, but partial cleavage also has been observed^4,5^. Not all degradation products are discarded and oligopeptides (3–15 amino acid residues) are provided for immunological processing and amino acids are recycled into newly synthesized proteins^6,7^.

Thousands of maternal-effect proteins are stored during oogenesis and degraded after fertilization by the UPS^8–10^. When degradation of maternal proteins is prevented by ablation of proteasome subunits, embryonic development is arrested or significantly delayed in early cleavage stages without affecting oogenesis^11,12^. These observations raise the possibility that cleavage by the UPS after fertilization may activate maternal proteins necessary for the reprograming oocytes into totipotent zygotes. However, there are few documented cases in which cleavage by the UPS is necessary for activation of biological activity^4,5^.

With these thoughts in mind, we sought to identified ubiquitin-regulated maternal proteins in 2-cell (2C) embryos and focus on transcriptional regulators or DNA/RNA-binding proteins. Individual proteins from an initial screen were expressed and processed with purified 20S proteasome from mouse ovaries. Several partially cleaved maternal proteins were detected and we focused on DPPA3 (developmental pluripotency associated 3, also known as Stella or PGC7), which is essential for normal mouse pre-implantation development. DPPA3 prevents demethylation by protecting 5-methylcytosine (5mC) of the maternal genome from TET3-mediated oxidation to 5-hydroxymethylcytosine (5hmC)^13–16^. However, TET3-mediated paternal 5mC oxidation is not essential for normal early mouse development^17,18^, which suggests additional function(s) for DPPA3 beyond epigenetic reprogramming. We now report an unanticipated cytoplasmic role for UPS-mediated DPPA3 polypeptide fragments in vesicle docking that involves interactions with subunits of the exocyst complex and maintenance of lysosomal membrane proteins. The absence of the DPPA3 cleavage products adversely affects pre-implantation development and female fertility.

## Results

### Identification of ubiquitin-regulated maternal DPPA3 protein

**Fig. 1:**
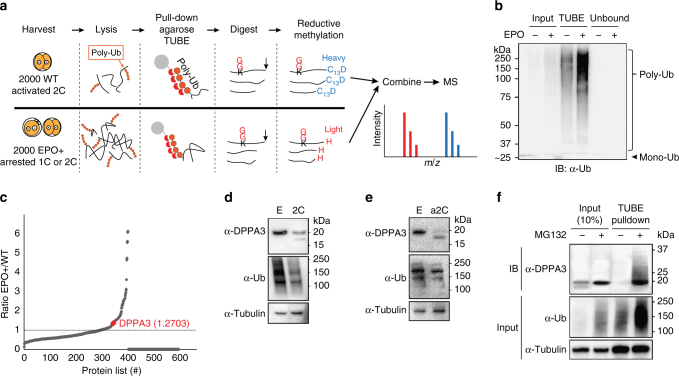
Identification of ubiquitinated DPPA3. **a** Schematic of procedures to identify ubiquitin-regulated maternal proteins. **b** Immunoblot with anti-ubiquitin antibody of poly-ubiquitinated maternal proteins isolated by agarose-TUBE before or after treatment with EPO. Molecular masses (kDa) on left. Ub, ubiquitin. **c** Dot plot of the ratio of identified maternal proteins before (WT) and after (EPO+) treatment with EPO as determined by stable isotope labeling and microscale tandem mass spectrometry. **d** Immunoblot of DPPA3 in ovulated MII eggs (E) and fertilized 2C embryos (2C) with anti-DPPA3 antibodies. Staining with anti-ubiquitin and anti-tubulin antibodies were used as load controls. Molecular masses (kDa) on right. **e** Same as **d** but with ovulated MII eggs (E) and parthenogenetically activated 2C embryos (a2C). **f** Immunoblot with anti-DPPA3 antibody of 2C lysates isolated using agarose-TUBE before or after treatment with MG132, a proteasome inhibitor. Staining with anti-ubiquitin and anti-tubulin antibodies were used as load controls. Molecular masses (kDa) on right

To confirm accumulation and degradation of poly-ubiquitinated maternal proteins, we performed immunoblots of ovulated MII eggs and parthenogenetically activated eggs treated with or without the proteasome inhibitor MG132 (Supplementary Fig. 1a). As expected, poly-ubiquitinated maternal proteins were degraded in activated eggs after 24 h at the 2C stage, but not if treated with MG132 (Supplementary Fig. 1b). To detect candidate maternal proteins, we isolated and parthenogenetically activated 4000 eggs. Half were treated with epoxomicin (EPO), a proteasome-specific inhibitor, and half were untreated controls. Ubiquitinated proteins were isolated from each sample with agarose-TUBEs (Tandem Ubiquitin Binding Entities) and differentially labeled with stable-isotope prior to microscale tandem mass spectrometry (Fig. 1a). Immunoblots documented that poly-ubiquitinated maternal proteins accumulated in EPO-treated activated eggs (Fig. 1b). A total of 627 ubiquitinated proteins were identified including known maternal proteins and those associated with the UPS (Supplementary Table 1).

Our initial studies focused on DPPA3, a maternal-effect protein implicated in protecting embryonic DNA from TET3-mediated demethylation and maternal genetic ablation of *Dppa3* results in cleavage-stage embryonic lethality^15^. DPPA3 was detected by mass spectrometry in the group of proteins that were degraded after activation (ratio 1.3, EPO+/WT) (Fig. 1c; Supplementary Fig. 1c, d). To localize endogenous DPPA3 in fertilized and parthenogenetically activated eggs, we used a polyclonal antibody and confocal microscopy (Supplementary Fig. 1e). DPPA3 was detected in the pronuclei and cytoplasm in fertilized 1C zygotes, less robustly in 2C embryos and only a faint signal was observed in the nuclei of 4C embryos. Similar observations have been made by others^13,19^. In parthenogenetically activated eggs, DPPA3 was present in the nuclei and cytoplasm in 1C zygotes, only in the cytoplasm of 2C embryos and was not detected in 4C embryos. Immunoblots of 300 ovulated MII eggs and 300 fertilized 2C embryos detected intact DPPA3 protein (~20 kDa) in both samples, albeit decreased in 2C embryos (Fig. 1d). Unexpectedly, a 17 kDa band was present in fertilized 2C embryos and parthenogenetically activated 2C eggs (Fig. 1d, e). As controls, the stability of DPPA3 was verified in MG132-treated 2C embryos and its poly-ubiquitination was confirmed by agarose-TUBE pulldowns (Fig. 1f). These combined studies suggested that maternal DPPA3 is poly-ubiquitination and is partially cleaved by the proteasome in early development.

### Partial cleavage of maternal DPPA3

**Fig. 2:**
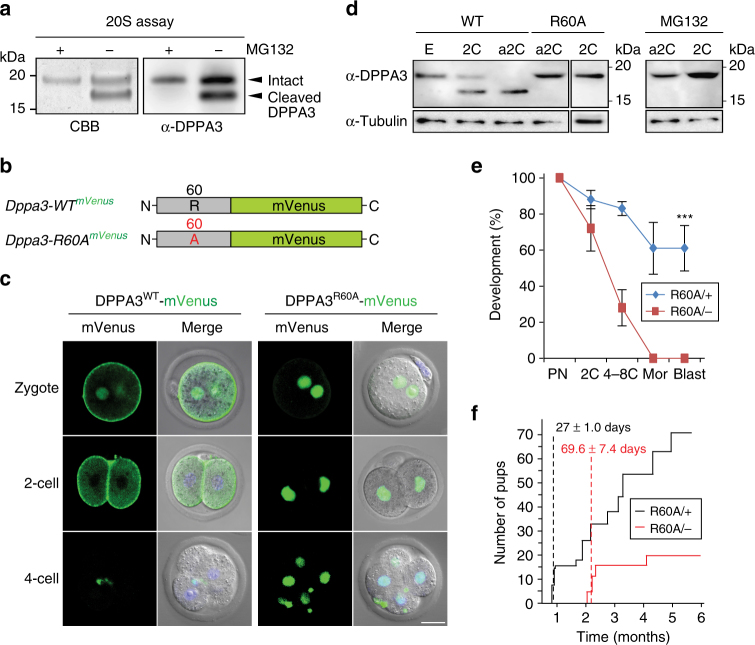
Partial cleavage of maternal DPPA3. **a** CBB staining of SDS-PAGE (left) and immunoblot with anti-DPPA3 antibody (right) of partially cleaved DPPA3 after in vitro digestion using recombinant DPPA3 and 20S proteasome purified from mouse ovaries in the presence and absence of MG132. Molecular masses (kDa) on left. **b** Schematics of cRNA encoding DPPA3^WT^ (top) or DPPA3^R60A^ (bottom) fused at their C-termini to mVenus that were microinjected into 1C zygotes. **c** cRNA encoding DPPA3^WT^ (left) and DPPA3^R60A^ (right) was injection into 1C zygotes and cultured to 2C, and 4C embryos prior to imaging with confocal microscopy alone and merged with DIC/Hoechst-stained images. Scale bar, 20 μm. **d** Immunoblots with anti-DPPA3 antibodies of embryo extracts from *Dppa3*^*WT*^ before (left) and after (right) treatment with MG132 and of embryo extracts from *Dppa3*^*R60A*^ (middle) mice. Staining with anti-tubulin antibodies was used as a load control. **e** Ovulated MII egg; 2C, fertilized 2C embryo; a2C, parthenogenetically activated 2C embryo. Molecular masses (kDa) on right. **e** Percentage of embryos at specified stage after parthenogenetic activation of eggs derived from *Dppa3*^*R60A/+*^ and *Dppa3*^*R60A/−*^ female mice. Error bars, s.d. from three replicates, ***Ρ < 0.001 by two-tailed Student’s *t*-test. **f** The number and time of live births from five female mice continuously mated for 6 months. Numbers indicate the time of the first litters, expressed as the number of days (avg. ± s.e.m.) after the start of mating

To verify partial cleavage of maternal DPPA3, we performed an in vitro 20S proteasome digestion assay. Recombinant DPPA3 was expressed in baculovirus and 20S proteasomes were purified from mouse ovaries by fast protein liquid chromatography (FPLC) (Supplementary Fig. 2a). Using mass spectrometry, we confirmed the absence of proteases in the purified 20S proteasome and determined comparable biological activity of 20S proteasome purified from either mouse ovaries or livers (Supplementary Table 2). Partial cleavage of DPPA3 between Arg60 and Asn61 was documented by microscale Edman degradation of the N-terminus of the cleaved recombinant protein (Fig. 2a; Supplementary Fig. 2b, c).

To determine cellular localization of WT (encoded by *Dppa3*^*WT*^) and DPPA3 mutated to prevent cleavage (encoded by *Dppa3*^*R60A*^), we microinjected cRNA encoding DPPA3 fused with fluorescent protein monomer Venus (mVenus) into fertilized 1C zygotes (Fig. 2b). DPPA3^WT^-mVenus was present in both male and female pronuclei and near the cell membrane of 1C zygotes (Fig. 2c). In 2C embryos, DPPA3^WT^ was absent from nuclei, but persisted near the plasma membrane before disappearing in 4C embryos. Strikingly, DPPA3^R60A^-mVenus was present only in the nuclei of 1C and 2C embryos where it persisted in 4C embryos. To confirm the localization and expression of DPPA3 fragments after cleavage, we injected cRNA encoding DPPA3^1–60^ and DPPA3^61–150^ fused with mVenus into 1C zygotes. The N-terminus of DPPA3 (1–60 aa) was primarily present in the cytoplasm and persisted to the morula stage of development. The C-terminus of DPPA3 (61–150 aa) was observed principally in the nucleus and disappeared from 4C embryos (Supplementary Fig. 2d). In germinal vesicle (GV) oocyte, DPPA3^1–150^ and DPPA3^1–60^ were primarily localized in the cytoplasm, whereas DPPA3^R60A^ and DPPA3^61–150^ were present only in the nucleus (Supplementary Fig. 2e). Thus, we conclude that cleavage of DPPA3 is needed for localization of the N-terminus in the cytoplasm during the first embryonic division.

DPPA3 is reported to be ubiquitinated at Lys96 (http://www.phosphosite.org/homeAction.action). To determine if the UPS was involved in the subsequent degradation of DPPA3, we generated a point mutation of Lys96 to prevent poly-ubiquitination (Supplementary Fig. 2f). Cellular localization of DPPA3^WT^-mVenus and DPPA3^K96A^-mVenus was not significantly different in 1C zygotes and 2C embryos, but DPPA3^K96A^-mVenus persisted in the cytoplasm of 4C embryos (Supplementary Fig. 2g). Furthermore, after treatment with MG132 to inhibit proteasomal degradation, DPPA3^WT^-mVenus also was observed adjacent to the plasma membrane in 1C zygotes (Supplementary Fig. 2h), and no cleavage of DPPA3^K96A^ was detected by immunoblot in transiently transfected cells (Supplementary Fig. 2i). These results indicate that cleavage of DPPA3 at ^59^LR^↓^NR^62^ is required for retention in the cytoplasm and that the subsequent degradation is mediated by ubiquitination of Lys96 and the UPS. Taken together, these observations raised the possibility of cytoplasmic function of DPPA3-derived polypeptides in early embryos.

### Preventing partial cleavage of maternal DPPA3

To genetically verify these observations, we used CRISPR/Cas9 to establish mouse lines expressing DPPA3^R60A^ and bred them to homozygosity (Supplementary Fig. 3a–d). Maternal DPPA3^WT^ was partially cleaved in 2C embryos from normal mating or after parthenogenetic activation of ovulated eggs (Fig. 2d). However, no cleavage was observed in 2C embryos or activated eggs derived from *Dppa3*^*R60A/R60A*^ female mice and MG132 prevented cleavage in activated eggs and fertilized 2C embryos (Fig. 2d and Supplementary Fig. 3e).

To determine the developmental potential of embryos derived from *Dppa3*^*R60A/−*^ mice, we performed in vitro culture assays with parthenogenetically activated eggs. Approximately 60% of activated eggs from *Dppa3*^*R60A/+*^ mice developed to blastocysts, but early embryonic development was significantly decreased in activated eggs from *Dppa3*^*R60A/−*^ mice and none progressed to blastocysts (Fig. 2e). During continuous mating with *Dppa3*^*−/−*^ male mice, *Dppa3*^*R60A/+*^ females (*n *= 5) delivered their first litters at 27 ± 1.0 days (avg. ± s.e.m.), whereas *Dppa3*^*R60A/−*^ females (*n* = 5) delivered their first litters at 69.6 ± 7.4 days. During the 6-month mating period, *Dppa3*^*R60A/−*^ female mice (*n* = 5) gave birth to substantially fewer litters (20) than *Dppa3*^*R60A/+*^ mice (>70) (Fig. 2f). Taken together, these results indicate that the presence of maternal DPPA3 in the cytoplasm and its partial cleavage is critical for normal embryonic development.

### Aggregation of endosomes in maternal DPPA3 deficiency

**Fig. 3:**
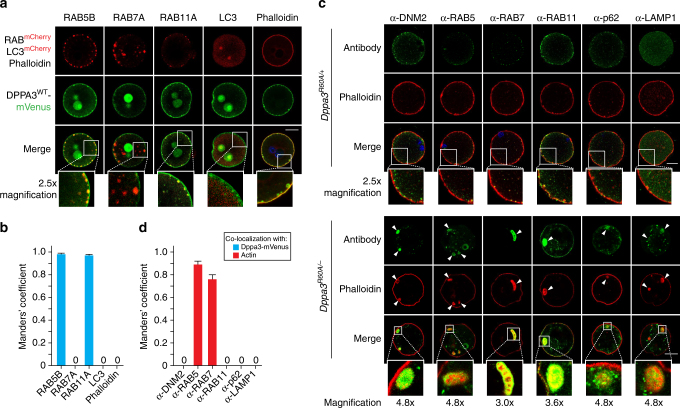
Partial cleavage of DPPA3 is critical for normal embryonic development. **a** Confocal images of RAB5B, RAB7A, RAB11A, and LC3 (top), DPPA3^WT^-mVenus (middle), and merged with Hoechst-stained images (bottom). cRNA encoding Dppa3^WT^-mVenus was co-injected with cRNA encoding each of the proteins tagged with *mCherry* into in vivo fertilized 1C zygotes. Phalloidin, stains F-actin. Insets, 2.5× magnification. Scale bar, 20 μm. **b** Manders’ co-localization coefficient of DPPA3^WT^-mVenus with other proteins tagged with *mCherry* imaged in **a**. **c** Confocal images of parthenogenetically activated and fixed eggs derived from *Dppa3*^*R60A/+*^ (upper) and *Dppa3*^*R60A/−*^ (lower) female mice and stained with antibodies to the indicated proteins and merged with Hoechst-stained images. Insets, 2.5–4.8× magnification. Arrowheads, aggregated vesicles. Scale bar, 20 μm. **d** Manders’ co-localization coefficient of actin (Phalloidin) and scissile vesicles (DNM2), early (RAB5), late (RAB7), recycling (RAB11) endosomes and lysosomes (p62, LAMP1) imaged in **c**. Error bars for **b**, **d**, s.e.m. from four replicates

DPPA3^WT^ was detected close to the plasma membrane in punctate foci (Fig. 2c). To identify proteins that might interact with cytoplasmic DPPA3 in early embryos, we selected several candidates associated with intracellular trafficking including RAB5 (early endosomes), RAB7 (late endosomes), RAB11 (recycling endosomes), and LC3 (autophagosomes)^20,21^. We then co-injected cRNAs of mCherry-tagged candidate proteins with cRNA encoding DPPA3^WT^-mVenus into parthenogenetically activated eggs. Cytoplasmic DPPA3 co-localized with early (RAB5B) and re-cycling (RAB11A) endosomes with Manders’ coefficients of 0.97–0.98 ± 0.01 (avg. ± s.e.m.), but not late endosomes (RAB7A) or autophagosomes (LC3) (Fig. 3a, b). We also observed that actin (stained with phalloidin) co-localized with early (RAB5) and late (RAB7) endosomes (Manders’ coefficients 0.89 ± .03 and 0.76 ± 0.04, respectively), but not with re-cycling (RAB11) endosomes, lysosomes (LAMP1, p62) or sessile vesicles (DNM2) (Fig. 3c, upper panel, d). Strikingly, vesicles containing each marker were abnormally massed as large globules that localized with F-actin in the cytoplasm of activated eggs isolated from *Dppa3*^*R60A/−*^ mice (Fig. 3c, bottom panel). Individual aggregates of actin co-localized with individual vesicular markers, which implies that each globule contains multiple and different endomembrane vesicles (i.e., there were relatively few actin aggregates that did not stain with a vesicular marker). The additional observation at higher magnification that vesicular markers were punctate and lack uniform co-localization with actin suggests spatial discontinuity by the inclusion of other vesicles of different origin in the large globules (Fig. 3c, lower panel). Thus, the observed globules in the absence of DPPA3 appear to contain mixtures of aggregated vesicles from the endomembrane system.

**Fig. 4:**
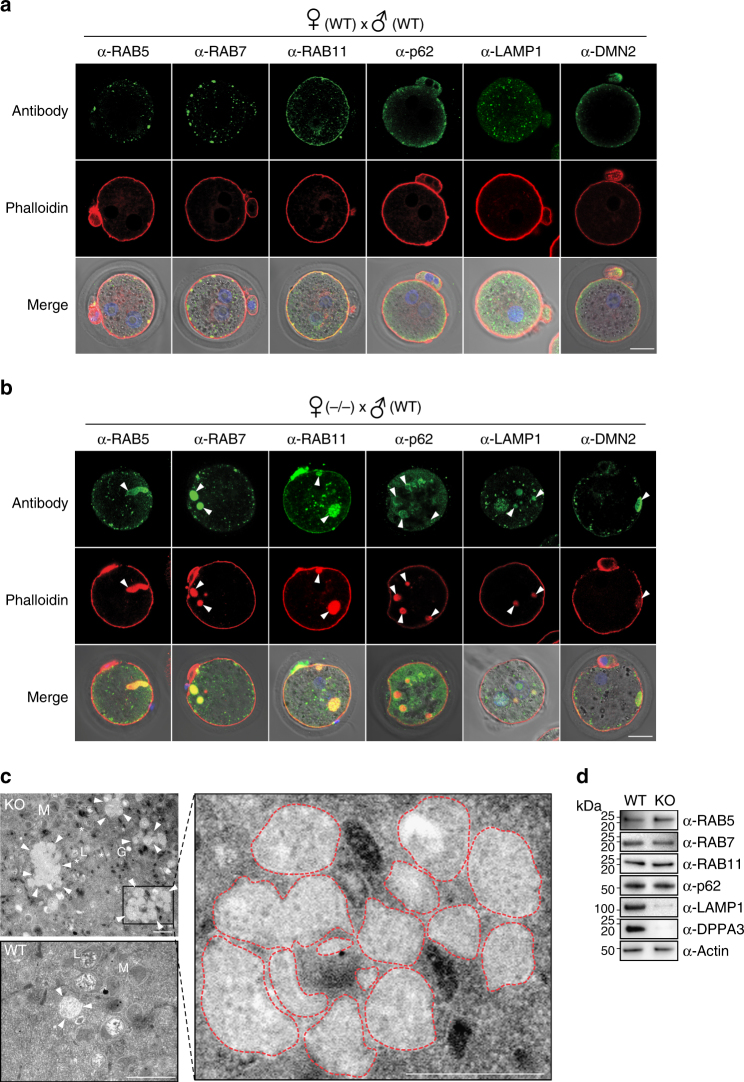
Abnormal aggregation of endosomes. **a** Confocal images of fixed 1C zygotes from maternal *Dppa3*^*WT*^ stained with antibodies to RAB5, RAB7, RAB11, p62, LAMP1, and DMN2 (top), phalloidin (middle), and merged with merged with DIC/Hoechst-stained images (bottom). Arrowheads, enlarged, aggregated vesicles. Scale bar, 20 μm. **b** Same as **a** but for *Dppa3*^*KO*^. **c** Electron microscopy of maternal *Dppa3*^*KO*^ (top) and *Dppa3*^*WT*^ (bottom) eggs with magnified fly-out on right. Arrowheads, multivesicular endosomes; L, lysosome; M, mitochondria; G, Golgi; asterisk, endoplasmic reticulum. Dotted red outline, vesicles. Scale bar, 500 nm. **d** Immunoblot of indicated proteins in ovulated MII eggs isolated from *Dppa3*^*WT*^ (left) and *Dppa3*^*KO*^ (right) mice

To further investigate associations with cytoplasmic DPPA3, we examined the localization of endosome-associated proteins (RAB5, RAB7, RAB11), lysosomal degradation-associated proteins (p62, LAMP1), and DMN2 (dynamin-associated microtubules) implicated in scission of new vesicles (Fig. 4a, b) in eggs after fertilization. In normal 1C zygotes, all assayed proteins were present in the periphery of the cytoplasm near the plasma membrane with puncta signals as expected. However, in 1C zygotes from *Dppa3*^*KO*^ mice, the proteins were abnormally aggregated in the cytoplasm where they co-localized with actin (Fig. 4b, arrowheads).

Clathrin and Cavoelin are involved in the earliest steps of endocytosis^22,23^. Therefore, we performed similar co-localization experiments using mCherry-tagged clathrin light chain (*Clc-mCherry*) and Cavoelin 1 (*Cav1-mCherry*) cRNA injected into WT eggs (Supplementary Fig. 4a) or antibody to each protein in zygotes from *Dppa3*^*WT*^ and *Dppa3*^*KO*^ female mice (Supplementary Fig. 4b, c). After cRNA injection into eggs, DPPA3 co-localized with CLC and CAV1 proteins at the plasma membrane, but not with CAV1 located more centrally in the cytoplasm (Supplementary Fig. 4a). In immunofluorescence analysis, clathrin heavy chain (CHC) and CAV1 were normally present in the periphery of the cytoplasm along with F-actin in *Dppa3*^*WT*^ 1C zygotes. However, in *Dppa3*^*KO*^ 1C zygotes, CHC (Supplementary Fig. 4b), but not CAV1 (Supplementary Fig. 4c), was present in the outer membranes of abnormally aggregated vesicles. Localized F-actin, which surrounds vesicles early in endocytosis^24^, was also observed and partially co-localized with DPPA3 (Supplementary Fig. 4d). This raised the possibility that partially cleaved maternal DPPA3 was involved in clathrin-mediated endomembrane trafficking.

We then quantified the size and number of abnormal aggregates in GV-intact oocytes, MII eggs, and 1C zygotes by staining with phalloidin (Supplementary Fig. 4e–g). The early endosome vesicles were approximately 0.5 μm in diameter in *Dppa3*^*WT*^, whereas abnormal aggregates were >5 μm in diameter in GV-intact oocytes and significantly increased (>20 μm) in MII eggs and 1C zygotes compared to corresponding stages in *Dppa3*^*KO*^ female mice (Supplementary Fig. 4f). Moreover, the number of abnormal aggregates over 5 μm in diameter was dramatically increased in maternal *Dppa3*^*KO*^ compared with *Dppa3*^*WT*^ eggs, but total amounts of aggregation in MII eggs and 1C zygotes were significantly decreased compared to GV-intact oocytes (Supplementary Fig. 4g). Most abnormal structures were localized close to the plasma membrane in mature oocytes and 1C zygotes, but not GV oocytes (Fig. 4b, arrowheads; Supplementary Fig. 4e). Taken together, these results suggest that membrane trafficking abnormalities included those involving clathrin during oocyte maturation and caused abnormal aggregation/positioning of vesicles in maternal *Dppa3*^*KO*^ mice.

### Increased accumulation of vesicles with DPPA3 deficiency

To verify the abnormal aggregation of endosomes in maternal DPPA3 deficiency, we imaged ovulated MII eggs isolated from *Dppa3*^*WT*^ and *Dppa3*^*KO*^ mice by electron microscopy. Although only single vesicles were observed in *Dppa3*^*WT*^ eggs, there were surprisingly large numbers of aggregated vesicles in *Dppa3*^*KO*^ eggs (Fig. 4c). This phenotype is quite like that described in double knockouts/downs of the major lysosome-associated membrane proteins, *Lamp1* and *2*^25,26^. Therefore, MII egg lysates from *Dppa3*^*KO*^ and *Dppa3*^*WT*^ eggs were analyzed by immunoblot. Although protein expression of RAB5, RAB7, RAB11, p62, and actin was not changed in the *Dppa3*^*KO*^ mice, LAMP1 and LAMP2 proteins were decreased in the absence of DPPA3 (Fig. 4d; Supplementary Fig. 5a, b). Dynamin 2 (DNM2), implicated in vesicle scission, was also decreased in *Dppa3*^*KO*^ mice, but protein levels of myosin IIa and myosin V associated with vesicular trafficking were not changed (Supplementary Fig. 5a, b). Using quantitative PCR, no significant decrease in abundance of mRNAs was observed in the *Dppa3*^*KO*^ mice (Supplementary Fig. 5c), indicating that changes were mediated post-transcriptionally. Thus, the absence of nuclear DPPA3 does not appear to affect the steady-state of endomembrane trafficking proteins raising the possibility that partially cleaved maternal DPPA3 promotes retention of LAMP1/2 markers of lysosomes and is important for vesicle trafficking. In support of this conceit, filipin staining documented accumulation of unesterified cholesterol in *Dppa3*^*KO*^ eggs as has been observed in *Lamp1/2* double knockout cells (Supplementary Fig. 5d)^25^. In addition, F-actin was abnormally aggregated in the cytoplasm of 1C zygotes treated with the PI3K inhibitor LY294002^27^ to prevent autophagy, but not by inhibition of actin polymerization with cytochalasin D or inhibition of proteasome activity with MG132 (Supplementary Fig. 5e). These observations are consistent with a role for DPPA3 in ensuring proper vesicle trafficking within the cytoplasm.

### DPPA3 binds to subunits of the exocyst complex

**Fig. 5:**
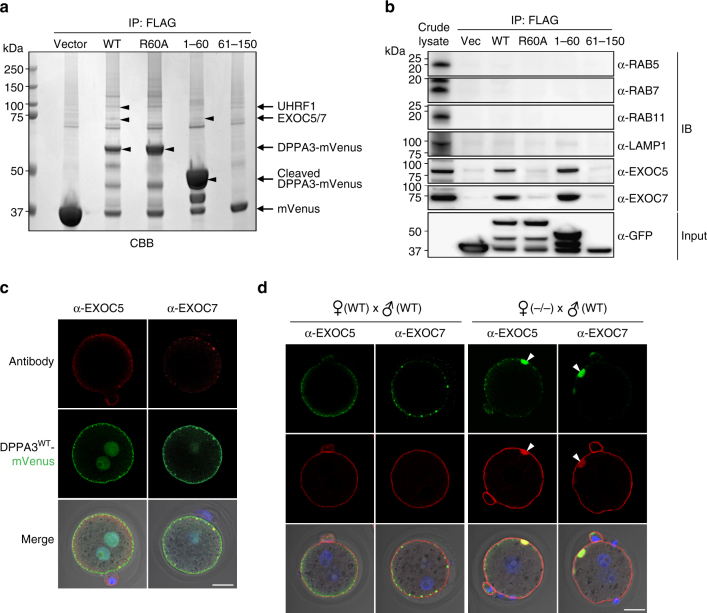
DPPA3 binds to subunits of the exocyst complex. **a** SDS-PAGE of 293T cell lysates immunoprecipitated with anti-FLAG antibodies after transfection with FLAG-tagged *mVenus* (control vector), *Dppa3*^*WT*^, *Dppa3*^*R60A*^, *Dppa3*^1–60^, and *Dppa3*^61–150^. CBB staining of UHRF1 and EXOC5/7 identified by LC-MS/MS. Molecular masses (kDa) on left. **b** Immunoblot after immunoprecipitation with anti-FLAG antibodies of 293T cell lysates transfected separately with FLAG-tagged *mVenus* (Vec), *Dppa3*^*WT*^, *Dppa3*^*R60A*^, *Dppa3*^1–60^, and *Dppa3*^61–150^. Antibodies used to probe blots on right. **c** Confocal images of subcellular localization of EXOC5 (top, left), EXOC7 (top, right), DPPA3^WT^-mVenus (middle), and merged with DIC/Hoechst-stained images (bottom). 1C zygotes were injected with DPPA3^WT^-mVenus, fixed and stained with antibody. Scale bar, 20 μm. **d** Confocal images of subcellular localization of EXOC5 and EXOC7 (top), phalloidin (middle), and merged with DIC/Hoechst-stained images (bottom) of fixed 1C zygotes derived from *Dppa3*^*WT*^ (left) and *Dppa3*^*KO*^ (right) female mice. Arrowheads, aggregated vesicles. Scale bar, 20 μm

To identify proteins that bind to DPPA3, we immunoprecipitated FLAG-tagged WT (1–150 aa), R60A (1–150 aa), 1–60 aa and 61–150 aa of DPPA3-mVenus transiently expressed in human embryonic kidney (HEK) 293T cells (Fig. 5a). Precipitated proteins were stained by Coomassie brilliant blue (CBB) (Fig. 5a) and protein bands between 75–100 kDa were excised for analysis by mass spectrometry. Among the identified proteins were UHRF1 (ubiquitin-like with PHD and ring finger domains 1) in WT, and EXOC5 and EXOC7 in WT and 1–60. DPPA3 was slightly polyubiquitinated by recombinant UHRF1 protein, but its polyubiquitination was increased when ovarian lysates were used which includes additional E3 ligases (Supplementary Fig. 5f). EXOC5 and EXOC7 are subunits of the 734 kDa exocyst complex (Fig. 5a; Supplementary Table 1), which is evolutionarily conserved and important for initial interactions between vesicles and target membranes via both proteins and lipids contacts^28,29^. The mouse exocyst contains eight proteins (EXOC1–8, corresponding to yeast Sec3, Sec5, Sec6, Sec8, Sec10, Sec15, Sec70, and Sec84, respectively^30^). EXOC1–4 form a core module that is connected to a subcomplex of the remaining vesicle-bound components that include EXOC5–8^31,32^. Genetic ablation of several subunits including *Exoc4* (*Sec8*) or *Exoc5* (*Sec10*) results in early embryonic lethality^33–35^.

Protein abundance of EXOC5 and EXOC7 was modestly decreased in ovulated MII eggs from *Dppa3*^*KO*^ mice (Supplementary Fig. 5a, b). FLAG-tagged full-length and the N-terminus (1–60) of DPPA3 bound EXOC5, EXOC6, EXOC7, and EXOC8, but not other subunits of the exocyst, endosome, and lysosome markers after transient expression in heterologous cells (Fig. 5b, Supplementary Fig. 5g). To confirm co-localization in fertilized eggs, we injected 1C zygotes with cRNA encoding DPPA3^WT^-mVenus and immunostained with antibodies to EXOC5 and EXOC7. Small puncta signals were observed in the periphery of the cytoplasm where they co-localized with DPPA3-mVenus (Fig. 5c). In addition, EXOC5 and EXOC7 abnormally aggregated with F-actin in maternal *Dppa3*^*KO*^ 1C zygotes (Fig. 5d). Taken together, these data suggest DPPA3 involvement in vesicle docking with an intact exocyst subcomplex in early embryos.

### Rescue with the N-terminus of DPPA3 or LAMP1/2

**Fig. 6:**
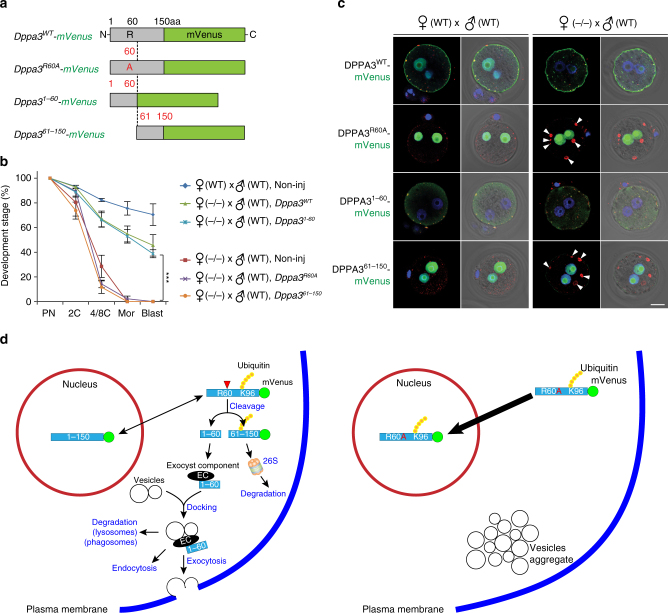
Rescue of pre-implantation embryos and model for maternal DPPA3 function. **a** Schematic of cRNA constructs with mVenus fused to the C-terminus of DPPA3^WT^, DPPA3^R60A^. DPPA3^1–60^ and DPPA3^61–150^. **b** Developmental progression of uninjected *Dppa3*^*WT*^ and *Dppa3*^*KO*^ 1C zygotes, or *Dpp3*^*KO*^ 1C zygotes injected with cRNA encoding DPPA3^WT^, DPPA3^1–60^, DPPA3^R60A^, and DPPA3^61–150^. Each data point reflects three independent biological samples each with >20 embryos. Error bars, s.d., ***P < 0.001 by two-tailed Student’s *t*-test. **c** Confocal alone and merged with DIC/Hoechst-stained images of 1C zygotes from *Dppa3*^*WT*^ (left) and *Dppa3*^*KO*^ (right) female mice injected with cRNA encoding DPPA3^WT^, DPPA3^R60A^. DPPA3^1–60^ and DPPA3^61–150^ fused to mVenus. Zygotes were fixed and stained with anti-RAB5 antibodies. Arrowheads, aggregated RAB5 endosomes. Scale bar, 20 μm. **d** DPPA3^WT^ is present in the nucleus and cytoplasm where it is poly-ubiquitinated and partially cleaved by the UPS at ^59^LR^↓^NR^62^. DPPA3^61–150^ is degraded while DPPA3^1–60^ binds to subunits of the exocyst complex to regulate intra-cytoplasmic vesicle trafficking (left). DPPA3^R60A^ cannot be cleaved and is detected only in the nucleus. The absence of DPPA3^1–60^ results in abnormal aggregation of vesicles in the cytoplasm and disrupts development (right). PN, pronuclei; C, cell; Mor, morula; Blast, blastocyst

To further investigate the role of DPPA3 in embryogenesis, we injected cRNA encoding intact, point mutated and truncated DPPA3 into maternal *Dppa3*^*KO*^ 1C zygotes, and observed in vitro pre-implantation development (Fig. 6a). Without injection, 70% of 1C zygotes from *Dppa3*^*WT*^ and 0% of 1C zygotes from *Dppa3*^*KO*^ female mice developed to blastocysts. Injection of cRNA encoding full-length DPPA3^1–150^ into 1C zygotes from *Dppa3*^*KO*^ female mice partially rescued the phenotype and ~45% progressed to blastocysts. Comparable results were obtained with DPPA3^1–60^, but neither DPPA3^61–150^ or DPPA3^R60A^ rescued *Dppa3*^*KO*^ embryos (Fig. 6b). In addition, the abnormal aggregates observed in *Dppa3*^*KO*^ 1C zygotes rapidly disappeared after injection of DPPA3^1–150^ or DPPA3^1–60^, but not DPPA3^R60A^ and DPPA3^61–150^ (Fig. 6c). These results indicate that partial cleavage of maternal DPPA3 by the UPS in the cytoplasm is important for normal endo/exocytosis and is essential for normal pre-implantation embryogenesis.

Because LAMP1 and LAMP2 proteins are decreased in *Dppa3*^*KO*^ eggs (Fig. 4d; Supplementary Fig. 5a, b), it was of interest to determine if their ectopic expression could rescue the null phenotype and promote development to blastocysts. cRNAs encoding LAMP1-FLAG and/or LAMP2-FLAG were injected into *Dppa3*^*KO*^ 1C zygotes, which were cultured in vitro. Over expression of LAMP1/2 was confirmed by immunoblot using polyclonal antibodies to each LAMP protein and monoclonal FLAG antibodies. The decreased levels of LAMP1/2 were significantly restored after injections into maternal *Dppa3*^*KO*^ 1C zygotes (Supplementary Fig. 6a). Surprisingly, the arrested phenotype was partially rescued by co-overexpression of both LAMP1 and LAMP2 whereas neither by itself had an affect (Supplementary Fig. 6b). We observed blastocyst formation in 28% of injected zygotes, which was less than observed with DPPA3^1–150^ (Fig. 6b; Supplementary Fig. 6b). Although the abnormal aggregates persisted in 1C zygotes after ectopic expression of LAMP1/2 (Supplementary Fig. 6c), they were not observed in 2C embryos (Supplementary Fig. 6d) and LAMP1/2 persisted in *Dppa3*^*KO*^ 1C zygotes after injection of cRNA encoding truncated DPPA3^1–60^ (Supplementary Fig. 6e). Thus, overexpression of LAMP1/2 is slower than DPPA3 in preventing the abnormalities and, although they partially rescue development, it is to a lesser extent than DPPA3.

### Reduced levels of 5hmC when DPPA3 is retained in pronuclei

DPPA3 binds to methylated histone H3 lysine 9 (H3K9me2), which is present predominantly, albeit not exclusively, in maternal chromatin^36^. This binding is reported to prevent TET3-mediated demethylation the first step of which is conversion of 5mC to 5hmC^37^. Because of its exclusive nuclear localization in 1C zygotes, we investigated if *Dppa3*^*R60A/−*^ would inhibit TET3 binding and affect the oxidation status of 5mC in male and female pronuclei.

To address this genetically, we performed localization assays using antibodies to DPPA3, TET3, and 5hmC in fertilized zygotes derived from *Dppa3*^*WT*^, *Dppa3*^*R60A/−*^, and *Dppa3*^*−/−*^ females after mating with *Dppa3*^*−/−*^ male mice (Supplementary Fig. 7). DPPA3 was present in the cytoplasm and pronuclei of *Dppa3*^*WT*^ zygotes and absent in *Dppa3*^*−/−*^ zygotes. In *Dppa3*^*R60A/−*^ zygotes, DPPA3 was strongly localized in both pronuclei, but absent from the cytoplasm (Supplementary Fig. 7). A small, but detectable, amount of TET3 and 5hmC was observed in the female pronuclei of *Dppa3*^*WT*^ and *Dppa3*^*−/−*^ zygotes compared to the much higher levels present in the male pronuclei. There was a significant decrease in levels of TET3 and 5hmC in both pronuclei of zygotes derived from *Dppa3*^*R60A/−*^ female mice, which suggests that the localization of uncleaved DPPA3 in the male and female pronuclei inhibits TET3 localization and decreases the observed amount of 5hmC, an oxidation product of 5mC.

## Discussion

DPPA3 is a maternal-effect factor present both in the nucleus and cytoplasm of eggs and 1C zygotes^13,38^. In this study, we found that DPPA3 is partially cleaved into a C-terminus polypeptide that is degraded and an N-terminus polypeptide (1–60 aa) present in the cytoplasm of the 1C zygote that facilitates vesicle trafficking in pre-implantation embryos (Fig. 6d). Abnormal aggregations of enlarged vesicles are observed in ovulated eggs and early embryos derived from *Dppa3*^*KO*^ and *Dppa3*^*R60A*^ female mice in which DPPA3 cannot be cleaved. We document that wild-type maternal DPPA3 is poly-ubiquitinated at Lys96 and partially cleaved at ^59^LR^↓^NR^62^ in the cytoplasm. DPPA3^61–150^ is degraded by the 26S proteasome, a process that is delayed by prevention of ubiquitination in DPPA3^K96A^ mutant mice. The N-terminus of DPPA3^1–60^ remains in the cytoplasm and we propose that it binds to the subcomplex (EXOC5-8) of the exocyst that forms on the vesicular surface of early (RAB5) and re-cycling (RAB11) endosomes and acts as a hub for endocytosis and exocytosis^39,40^. In the absence of DPPA3, early and re-cycling endosomes enlarge and aggregate, but this does not prevent their maturation as they accumulate late endosomes (RAB7) and lysosomes (LAMP1/2) markers. The phenotype is not dissimilar to that observed after *Lamp1/2* knockdowns in early mouse embryos with siRNA^26^. Together, these data document a cytoplasmic function for partially cleaved maternal DPPA3 in vesicular trafficking that is essential for early embryogenesis.

The UPS is a tightly regulated process in which attachment of ubiquitin to a target protein serves as signal for degradation^41,42^. Ubiquitination occurs when an E3 ligase enzyme binds to both substrate protein and an E2 thio-esterified with ubiquitin, bringing them in proximity so that the ubiquitin is transferred from the E2 to the target protein^43,44^. Some E3 ligases, such as PARKIN, can bind multiple substrates through different functional domains^45^. The majority of mono/poly/multi-ubiquitinated proteins are targeted for degradation by the 26S proteasome^46^, whereas the 19S proteasome complex recognizes misfolded proteins in an ATP-dependent manner and transfers them into the inner catalytic chamber of the 20S proteasome.

Our results document that DPPA3 is regulated by poly-ubiquitination of Lys96, suggesting involvement of a specific E3 ligase. In a binding assay, DPPA3 bound UHRF1 (ubiquitin-like PHD and RING finger 1 protein, also known as ICBP90 and Np90), a multi-domain protein that acts as a key epigenetic regulator that bridges DNA methylation and chromatin modification^47–49^. UHRF1 includes a RING (Really Interesting New Gene) finger domain in the C-terminal region that is required for ubiquitin ligase activity. In addition to its reported modification of histone H3^50^, the interactions with the C-terminus of DPPA3^51^ suggests that UHRF1 may ubiquitinate maternal DPPA3 to facilitate partial cleavage by the UPS. There are a few examples where the proteasome stops degradation part way through a polypeptide chain and releases a partially degraded polypeptide. This partial degradation occurs when an internal ubiquitination site or another motif encoded in the substrate signals arrest of the UPS^52^. Although we could not define a physiological cause for the partial cleavage of maternal DPPA3 between Arg60 and Asn61, this region of the protein might be folded to define a unique architectural feature that stops the UPS and results in partial degradation of DPPA3.

Maternal proteins and organelles accumulate during oogenesis and are stored in preparation of the egg-to-embryo transition. Egg activation, triggered by sperm entry, initiates degradation of maternal proteins and remodeling of cytoplasmic organelles^53^. The two major mechanisms of proteolysis are the UPS and the lysosomes^3,41,42^. The former selectively degrades normal and abnormal proteins as described above. Lysosomes, along the autophagy, are the principal mechanism to degrade proteins with long half-lives, organelles, and large protein aggregates^54,55^. In some cases, the endocytic pathway delivers extracellular material and plasma membrane constitutes to lysosomes under direction of specific targeting signal^24^.

Our results of *Dppa3*^*KO*^ and *Dppa3*^*R60A*^ mice document decreased abundance of LAMP1/2 proteins with abnormal cytoplasmic organelles and arrested embryogenesis at the 4C or 8C stage similar to the phenotypes of a double *Lamp1/2* knockdown and autophagy-deficient zygotes^26^. Numerous early/initial autophagic vacuoles and late/degradative endosomes were observed in the cytoplasm of double *Lamp1/2* knockdown embryos^25^. Consistent with the similar phenotypes, the arrested early embryonic development of *Dppa3*^*KO*^ was partially rescued by overexpressing LAMP1/2, although not as effectively as overexpression of DPPA3^WT^. Taken together, these results suggest that DPPA3 could be an upstream regulator of critical pathways for major protein degradation in pre-implantation development. In the absence of documented direct binding of DPPA3 with LAMP1/2, these effects are likely to be indirect and involve yet to be identified intermediates.

DPPA3 was first identified as a primordial germ cell (PGC) and early pre-implantation embryo marker in the mouse^38,56^. Unexpectedly, genetic ablation in female mice did not affect germ cell development, but did prevent early embryogenesis. Homozygous null females mated with WT males had reduced fertility because early embryos underwent precocious compaction and failed to develop into blastocysts^13,14^. Thus, the arrested pre-implantation development was not rescued by the embryonic expression of *Dppa3* and maternal DPPA3 is essential for early embryogenesis. Normally, levels of oxidized 5hmC increase in 1C zygotes as TET3 methylcytosine dioxygenase oxidizes 5mC to 5hmC, but only the paternal genome is demethylated^57^. The protection of the female pronucleus has been ascribed to maternal DPPA3 binding to di-methylated histone 3 lysine 9 (H3K9me2) and inhibiting access of the TET3 methylcytosine oxidase^37^. Consistent with this model, both the maternal and paternal genomes of zygotes are demethylated in mice lacking maternal DPPA3^37^.

DPPA3 was present in the pronuclei and cytoplasm of 1C zygotes derived from *Dppa3*^*WT*^ female mice, in the pronuclei, but not the cytoplasm of 1C zygotes from *Dppa3*^*R60A/−*^ female mice and was not detected in *Dppa3*^*−/−*^ zygotes. TET3 was present in the paternal and maternal pronuclei of 1C zygotes from *Dppa3*^*WT*^ and *Dppa3*^*KO*^ females, but its intensity was significantly decreased in 1C zygotes derived from *Dppa3*^*R60A/−*^ female mice. Consistent with a model that 5hmC in paternal pronuclei is generated by pre-existing TET3 in the egg, 5hmC was also significantly decreased in *Dppa3*^*R60A/−*^ zygotes. These observations agree with previous reports that maternal DPPA3 acts to protect the maternal genome from demethylation in fertilized 1C zygotes^37^. However, it has recently been reported that embryos from homozygous *Tet3*^*Null*^ female mice mated with WT males develop normally and oxidation of 5mC to 5hmC was detected in both pronuclei^17,18^. Additional recent studies report that decreased levels of 5mC do not results in reciprocal increases in oxidation products (5hmC, 5meC), which raises further questions as to the role of DPPA3 in zygotic demethylation^58,59^. Thus, rather than a nuclear function, the cytoplasmic role of cleaved DPPA3 in regulating intracellular trafficking may be of greater import for successful cleavage-stage development.

## Methods

### Mouse husbandry and isolation of eggs and embryos

Mice were maintained in compliance with the guidelines of the Animal Care and Use Committee of the National Institute of Health under a Division of Intramural Research, NIDDK-approved animal study protocol. Eggs and embryos were collect in M2 medium (Zenith Biotech), embryos were cultured in KSOM medium (Zenith Biotech) at 37 °C in 5% CO_2_^9,60^.

### Identification of DPPA3

Four thousand eggs were collected from the oviducts of 8–12-week-old females that had been induced to ovulate with 5 IU equine chorionic gonadotropin followed by 5 IU human chorionic gonadotropin (hCG) 48 h later. Eggs were collected approximately 16 h after hCG injection, placed in CaCl_2_-free M16 medium and treated with 0.1% hyaluronidase (Sigma) until the cumulus cells dispersed. The eggs were then activated in CaCl_2_-free M16 medium supplemented with 5 mM SrCl_2_ (Sigma) at 37 °C in 5% CO_2_^61^. After 6 h incubation, half were treated with 1 μM EPO (Boston Biochem), a proteasome-specific inhibitor, and half were untreated controls. Following an overnight culture, ubiquitinated proteins were isolated from each sample with agarose-TUBEs (Tandem Ubiquitin Binding Entities, LifeSensors) and differentially labeled with stable-isotope prior to analysis by microscale tandem mass spectrometry^62^.

The proteins were non-specifically dissociated from TUBE using 100 mM Tris, 8 M urea acidified to pH 3.5 with formic acid twice for 30 min at 37 °C. After adjusting the pH to 8 using small aliquots of ammonium hydroxide, the pooled sample was reduced with 10 mM DTT (37 °C, 30 min), alkylated with 30 mM iodoacetamide (room temperature, 1 h, in the dark) and any remaining reactive iodoacetamide was scavenged by adding 30 mM β-mercaptoethanol (room temperature, 30 min). Lys-C was added (2 μg, WAKO) and the samples were digested overnight at room temperature. The samples were then diluted with three parts of 100 mM ammonium acetate and digested with the addition of trypsin (2 μg, Promega) for 4 h. After acidification with formic acid, each sample was applied to an in-house fabricated C18 STAGE tip^63^ containing four layers of Empire C18 reverse phase material and loaded using centrifugal force (300 × *g*). After loading was complete, each column was washed with 200 μl of 1.6% formic acid and then 100 μl of plain water. Reductive dimethylation labeling^64,65^ of peptides on the STAGE tips was performed by labeling the control with the 2× deuterium methyl groups and the EPO-treated sample with normal methyl groups^62^. After labeling, the column-bound peptides were washed with 200 μl 1.6% formic acid and then 2 × 200 μl 100 mM ammonium acetate in 1.6% formic acid with a final spin to remove excess liquid from the column. Peptides were eluted from each column using 100 μl of 0.4% formic acid, 40% acetonitrile followed by a further 100 μl of 0.4% formic acid 80% acetonitrile. The sub-samples were combined and mixed thoroughly before being applied to an in-house made STAGE tip containing four layers of Empire SCX using centrifugal force. After loading, the column was washed with 2 × 350 μl of 0.4% formic acid, 40% acetonitrile and then 2 × 350 μl of 0.4% formic acid, 80% acetonitrile. Next, the sample was eluted with 100 μl 0.4% formic acid, 20% acetonitrile, 500 mM ammonium acetate and the 100 μl 0.4% formic acid, 50% acetonitrile, 200 mM ammonium acetate. After reducing the sample to a partially dry salt cake by warming to 50 °C with dry nitrogen, the sample was re-suspended in 30 μl of 0.8% formic acid. Data were collected^66^ and analyzed with MaxQuant^67^ (Supplementary Data 1).

### Recombinant DPPA3 expression and purification

Mouse *Dppa3* (Addgene #13365) was cloned using PCR from cDNA templates for baculovirus expression^68^. cDNA encoding wild-type and mutant *Dppa3* was cloned into the pFastBac-HT-A expression vector (Invitrogen) containing an N-terminal 6xHis tag. Recombinant proteins were expressed in Sf9 insect cells (Invitrogen), purified by 6xHis/Ni-NTA (Qiagen) and assayed on SDS-PAGE. Uncropped images are in Supplementary Figs. 8–12.

### Purification of 20S proteasome for in vitro digestion assay

Mouse ovaries (60) and liver controls (10) were collected and homogenized in three volumes of 25 mM Tris-HCl buffer (pH 7.4) containing 1 mM DTT and 0.25 M sucrose in a Potter-Elvehjem homogenizer^69,70^. Nucleus and cytoplasmic organelles were removed from total lysates by ultracentrifugation and 20S proteasomes were purified from cytosolic lysates using an AKTA purifier FPLC (GE Healthcare) with ion-exchange (Resource Q 1 ml, GE Healthcare), gel filtration (Superdex 200 10/300 GL, GE Healthcare), and hydroxyapatite (Bio-Scale CHT2-I, Bio Rad) columns. After each FPLC, 20S proteasomes activity was tested by peptidase activity using a fluorescent peptide substrate, succinyl-Leu-Leu-Val-Tyr-7-amino-4-methylcoumarin (Suc-LLVY-AMC, Peptide Institute)^69^ in a low concentration of SDS (0.025% (w/v)) for artificial activators of latent 20S proteasomes. The presence of all α and β subunits of 20S proteasome and the absence of endo/exogenous proteases were confirmed by LC-MS/MS (Livers (H)/Ovaries (L)) analysis (Supplementary Data 2).

Recombinant DPPA3 protein was denatured, reduced, and S-carboxymethylated^70^. Proteins were dialyzed against 0.1 M Tris-HCl (pH 8.0) and stored at −80 °C. DPPA3 (5 μg) mixed with proteasomes (1 μg) and incubated (2 h, 37 °C) with or without MG132 (10 μM, Sigma), a proteasome inhibitor. Partial cleavage of DPPA3 was confirmed by CBB (Thermo) staining and immunoblot probed with polyclonal anti-DPPA3 antibody (Abcam) or monoclonal anti-His antibody (Santa Cruz Biotechnology).

### N-terminal protein sequencing

After in vitro digestion of 100 μg of recombinant DPPA3, cleaved peptides were separated in SDS-PAGE and transferred to polyvinylidene difluoride (PVDF) membrane (Invitrogen). The membrane was stained with the Pierce Reversible Protein Stain Kit for PVDF membranes (Thermo). The cleaved bands were isolated in ultrapure water and analyzed by N-terminal protein sequencing (Facility for Biotechnology Resources, CBER, FDA).

### Generation of *Dppa3*^*R60A/R60A*^ mice

B6D2_F1_ (C57LB/6 × DBA2) female mice and ICR mouse strains were used as embryo donors and foster mothers, respectively. Hormonally stimulated B6D2_F1_ female mice (8–12 week old) were mated to B6D2_F1_ male mice (~1 year old) and fertilized embryos were collected from oviducts. *Cas9* mRNAs (Addgene #42251, 100 ng/μl), sgRNA (50 ng/μl), and donor ssDNA oligonucleotides (100 ng/μl) were mixed and injected into the cytoplasm of fertilized 1C zygotes in M2 medium. Injected zygotes were cultured in KSOM (37 °C, 5% CO_2_) for 3.5 days to the blastocyst stage. Blastocysts (15–20) were transferred into the uteri of pseudopregnant ICR female mice at E2.5.

### Electron microscopy

Ovulated eggs isolated from wild-type and *Dppa3*^*KO*^ female mice were fixed in 2% glutaraldehyde in 0.1 M cacodylate buffer (pH 7.4) and processed for electron microscopy^71^.

### RNA isolation and quantitative real-time RT-PCR

Total RNA was isolated from 50 GV oocytes using NucleoSpin RNA (Macherey-Nagel) and cDNA was synthesized using PrimeScript™ RT Master Mix (Takara Bio)^9^. Quantitative RT-PCR was performed using SYBR Green Master Mix (Takara Bio) and QuantStudio 6 Flex Real-Time PCR System (Thermo). The primers are listed in Supplementary Table 2.

### Immunoprecipitation

Wild-type, R60A, 1–60, 61–150, and K96A isoforms of *Dppa3* cloned into *pcDNA4-mVenus* (Addgene #44118) were transiently expressed in HEK 293T cells. Cells were scrapped off the plates and centrifuged (800 × *g*, 5 min). After being washed twice with cold 1× PBS(-), cells were lysed (15 min on ice) in IP buffer (50 mM Tris, pH 7.4, 150 mM NaCl, 10% glycerol, 1% Triton X-100, 0.5 mM EDTA, 10 mM NaF, 100 μM orthovanadate, 200 μM PMSF) supplemented with a protease inhibitor cocktail (Roche). The cell lysates were clarified by centrifugation (15,000 × *g*, 4 °C, 15 min) and the supernatant incubated with anti-FLAG M2 affinity Gel (Sigma) at 4 °C with rotation. The complexes were precipitated, washed twice with IP lysis buffer supplemented with 500 mM NaCl, then six times with IP lysis buffer alone. Bound complexes were eluted with FLAG peptide (100 μg/ml) (Sigma) and separated by SDS-PAGE (10%) after boiling (5 min, 95 °C) in 1× SDS sample buffer. CBB-stained bands in the 75–100 kDa region of gel were isolated and identified by LC-MS/MS (Taplin Mass Spectrometry Facility, Harvard Medical School).

### Immunoblots

Proteins were separated on 8, 10, and 4–12% SDS gels (Invitrogen) and electrophoretically transferred to PVDF membranes (Invitrogen). The membranes were incubated (20 min, RT) in Blocking One (Nacalai Tesque), washed with Tris-buffered saline containing 0.1% Tween 20 (TBST) and incubated (4 °C, overnight) with primary antibodies. The membranes then were washed in TBST and incubated (1 h, RT) with secondary antibodies, washed with TBST and developed using Amersham ECL Western Blotting Detection Reagent (GE Healthcare). The antibodies are listed in Supplementary Tables 3 and 4.

### In vitro ubiquitination assay

6xHis-tagged recombinant proteins for UBE1, E2 (Ubc4 and Ube2e), Ubiquitin, UHRF1 (Addgene #28163), and DPPA3 were purified using baculovirus and *E. coli* expression systems. Ubiquitination assay were then performed in the presence of 250 μg UBE1, 500 μg E2s, 500 μg E3, 15 μg ubiquitin, 500 ng DPPA3 as a substrate and UB buffer (2 mM ATP, 1 mM DTT, 50 mM Tris, pH 7.4, 2 mM MgCl_2_), and incubating at 37 °C for 1 h. 5 μg of ovarian tissue lysed by lysis buffer (50 mM Tris, pH 7.4, 150 mM NaCl, 10% glycerol, 0.5% NP-40, 0.5 mM EDTA, 10 mM NaF, 100 μM orthovanadate, 200 μM PMSF) were used as a positive control. Reactions were stopped by the addition of 1× sample buffer and boiling and the reaction mixtures were analyzed by immunoblotting.

### Immunocytochemistry and imaging

Collected eggs and embryos were fixed (15 min, RT) in 4% paraformaldehyde (Electron Microscopy Sciences) diluted by phosphate-buffered saline (PBS, Gibco) and permeabilized (20 min, RT) by PBS containing 0.2% Triton X-100 (AmericanBio). Samples were incubated (4 °C, overnight) with primary antibodies in PBS containing 30 mg/ml bovine serum albumin (Millipore). Samples were then incubated (1 h, RT) with Alexa Fluor secondary antibodies and phalloidin (Invitrogen). Specimens were mounted on glass slides in PBS containing 10 μg/ml Hoechst (Invitrogen). To stain 5hmC, in vivo mated zygotes were isolated and incubated (30 min, RT) in 4 N HCl and then incubated (30 min, RT) in 0.1 M EDTA. Fixed samples were imaged by LSM 780 (Carl Zeiss) confocal microscopy^71^ and Manders’ co-localization coefficients^72^ were calculated by LSM Image Examiner.

### In vitro cRNA synthesis and microinjection

mVenus and mCherry-tagged *Dppa3* and candidate cDNAs were cloned into pcDNA3.1 expression vector (Invitrogen), transcribed with mMESSAGE mMACHINE T7 ULTRA Transcription Kit (Ambion), purified using MEGAclear Transcription Clean-Up Kit (Ambion) and stored no more than 3 months at −80 °C. Synthesized cRNA (300 ng/μl) was microinjected into in vivo fertilized 1C zygotes in M2 medium. 500 ng/μl cRNAs were used to rescue mutant 1C zygotes. Microinjected cells were transferred to KSOM medium, incubated for 3 h to select embryos positive for mVenus and mCherry fluorescence, which were cultured at 37 °C, 5% CO_2_ until use or the blastocyst stage (3.5 days).

### Data availability

The data that support the findings of this study are available from the corresponding author on request.

## Electronic supplementary material

Supplementary Information

Description of Additional Supplementary Files

Supplementary Data 1

Supplementary Data 2
