# Supplementary material for: Cytoplasmic cleavage of DPPA3 is required for intracellular trafficking and cleavage-stage development in mice
Source: Nat Commun. 2017 Nov 21;8:1643. doi: 10.1038/s41467-017-01387-6 (PMC5696369; doi:10.1038/s41467-017-01387-6)
Supplement: Supplementary file 1 — Supplementary Information [file 41467_2017_1387_MOESM1_ESM.pdf]

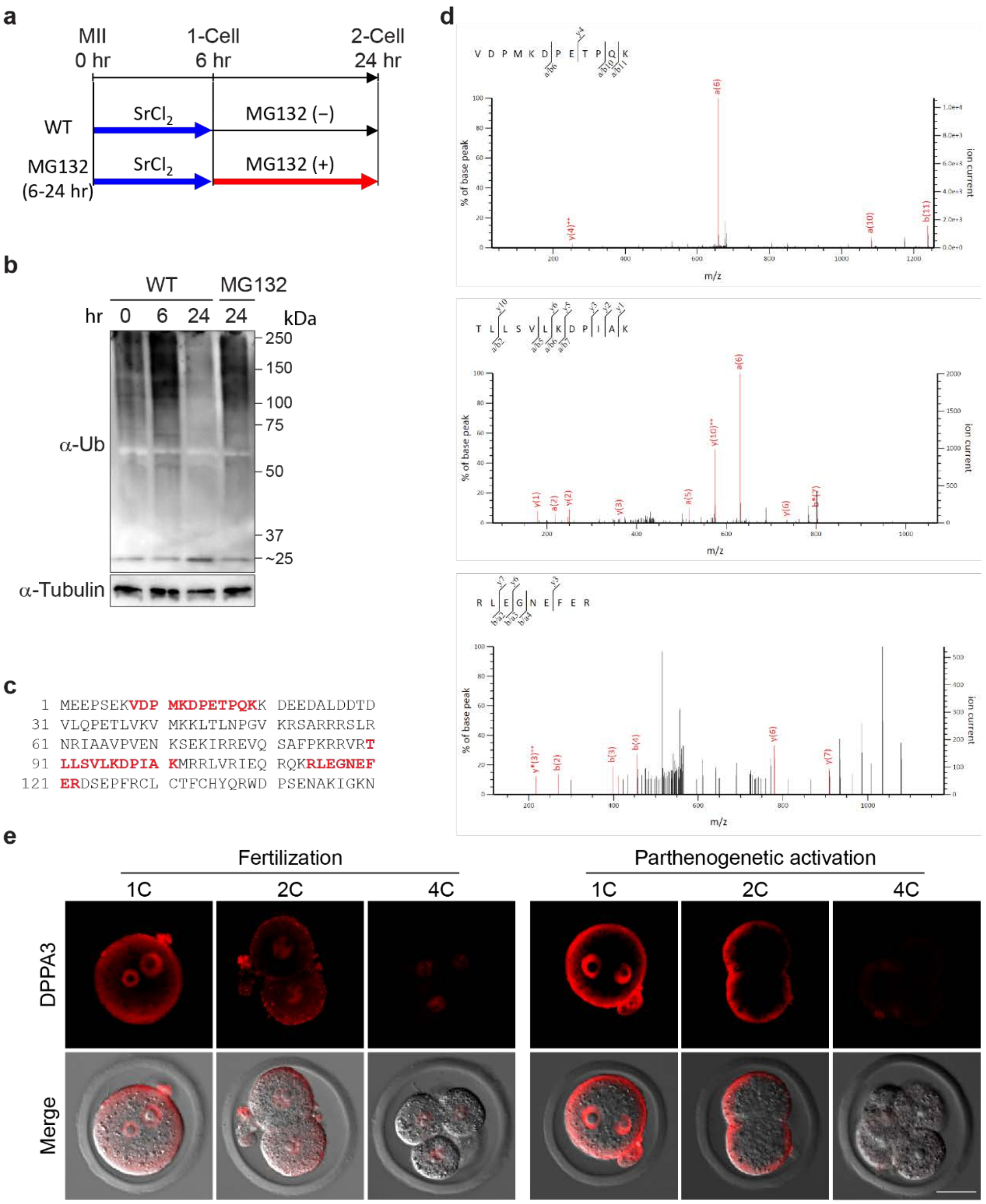

Supplementary Figure 1 | Poly-ubiquitination of maternal proteins and identified DPPA3. (**a**) Schematic of egg activation with $SrCl_2$ for 6 hr followed by *in vitro* culture for an additional 18 hr with or without MG132 to block the degradation of poly-ubiquitinated maternal proteins. (**b**) Immunoblot of poly-ubiquitinated maternal proteins at indicated time points probed with anti-ubiquitin antibody. Tubulin provided a loading control. Molecular mass (kDa) on right. (**c**) Amino acid sequence of DPPA3 with

matched peptides from microscale MS/MS in bold red. (**d**) MS/MS fragmentations of each identified peptide. (**e**) Confocal microscope images of endogenous DPPA3 stained with antibody alone (top) and merged with DIC images (bottom) of fertilized 1-cell (1C), 2C and 4C embryos (left) and parthenogenetically activated 1C, 2C and 4C embryos. Scale bar, 20 μm.

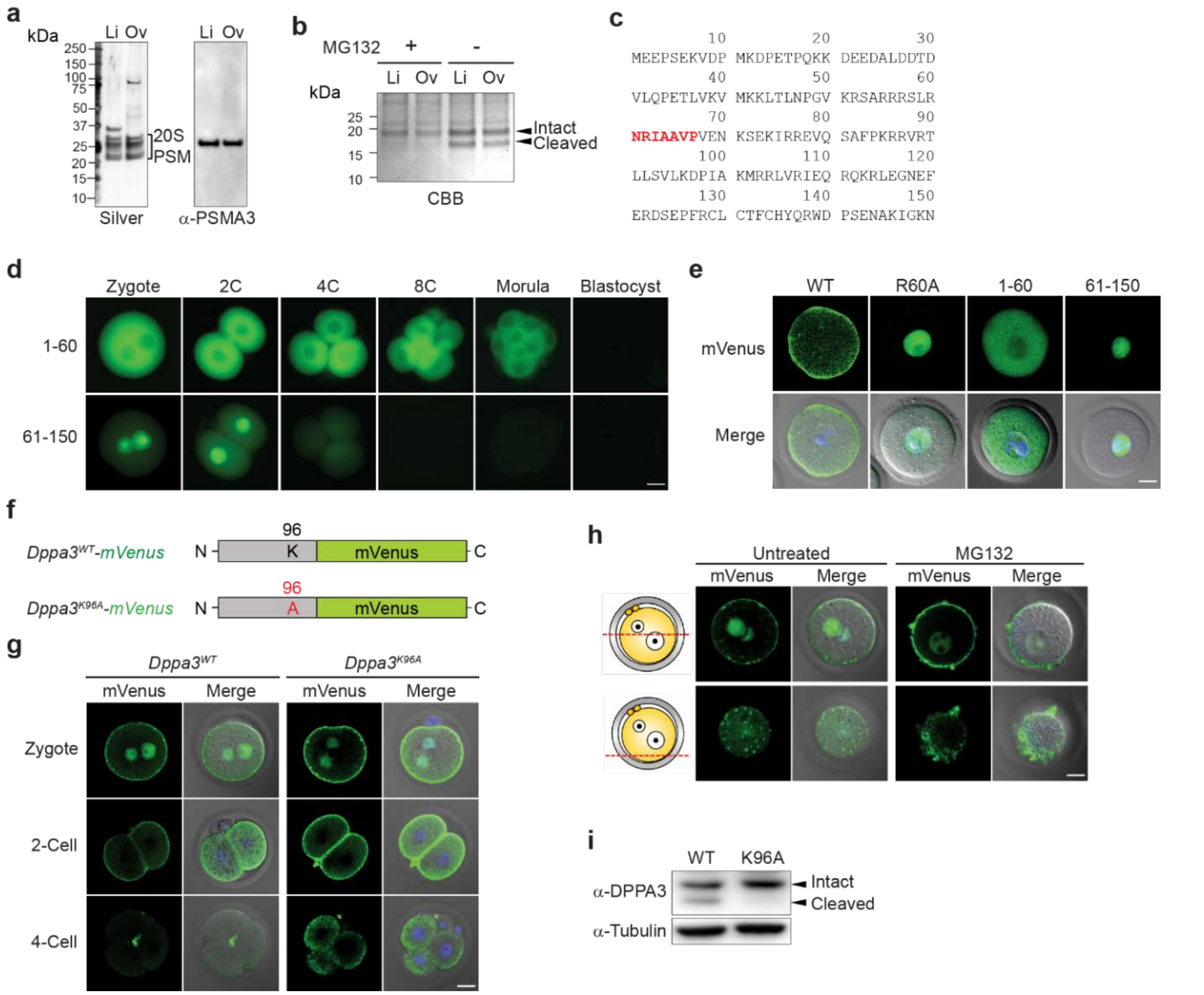

Supplementary Figure 2 | Partial cleavage of DPPA3. (**a**) 20S proteasomes (PSM) from mouse liver (Li) and ovary (Ov) were separated by SDS-PAGE and visualized by silver staining (left) and immunoblot (right) using polyclonal anti-PSMA3 (α7) antibody. Molecular mass (kDa) on left. (**b**) Coomassie Brilliant Blue (CBB) staining of partially cleaved DPPA3 after *in vitro* digestion using recombinant DPPA3 and 20S proteasome purified from mouse livers and ovaries with or without proteasome inhibitor MG132. Molecular mass (kDa) on left. (**c**) Amino acid sequence of DPPA3 with Edman degradation N-terminal sequence of 17 kDa cleavage fragment in bold red. (**d**) cRNA encoding DPPA3$^{1\text{-}60}$ (top) and DPPA3$^{61\text{-}150}$ (bottom) was injection into 1C zygotes and cultured to blastocysts prior to imaging with confocal microscopy. Scale bar, 20 µm. (**e**) Confocal alone and merged with DIC/Hoechst stained images in GV oocytes after microinjection of *Dppa3^{WT}* (WT), *Dppa3^{R60A}* (R60A),

Dppa3$^{1\text{-}60}$ (1-60) and *Dppa3*$^{61\text{-}150}$ (61-150) cRNA. Scale bar, 20 μm. (**f**) Schematic of microinjected cRNA of *Dppa3*^{WT} and *Dppa3*^{K96A} that cannot be ubiquitinated fused with mVenus. (**g**) Confocal alone and merged with DIC/Hoechst stained images after microinjection and culture of 1C zygotes from *Dppa3*^{WT} (left) and *Dppa3*^{K96A} (right) female mice to 2C and 4C. Scale bar, 20 μm. (**h**) Middle (top) and bottom (bottom) optical confocal sections of 1C zygotes from *Dppa3*^{WT} female mice either treated (right) or untreated (left) with 10 μM MG132. Scale bar, 20 μm. (**i**) Immunoblot of DPPA3^{WT} and DPPA3$^{\mathrm{K96A}}$ transiently expressed in HEK 293T cells using polyclonal anti-DPPA3 antibody. Tubulin was used as a load control.

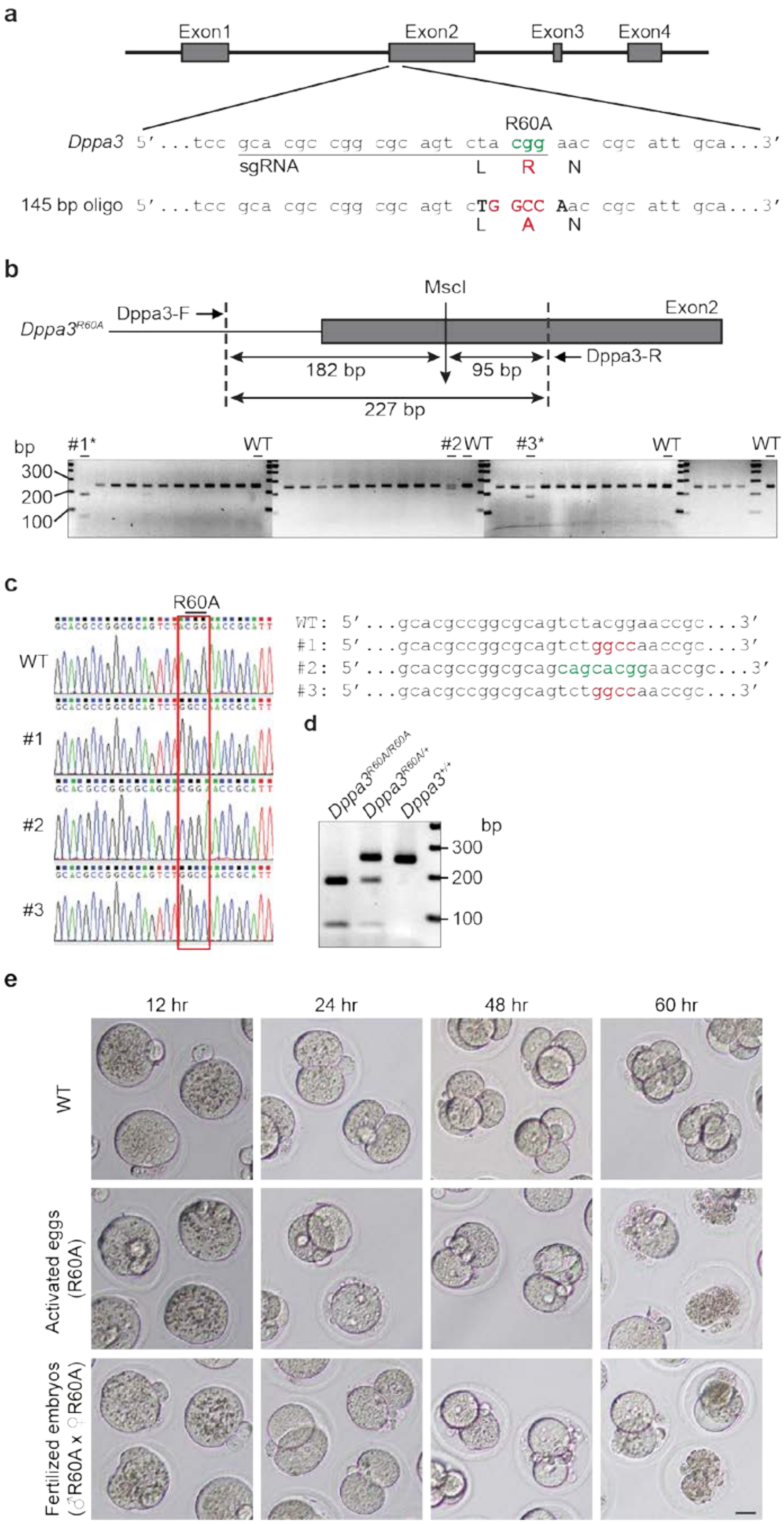
a
Exon1
Exon2
Exon3
Exon4
R60A
Dppa3 5'...tcc gca cgc cgg cgc agt cta cgg aac cgc att gca...3'
sgRNA
L R N
145 bp oligo 5'...tcc gca cgc cgg cgc agt cTG GCC Aac cgc att gca...3'
L A N
b
MscI
Dppa3-F
Exon2
Dppa3R60A
182 bp
95 bp
Dppa3-R
227 bp
bp
#1*
WT
#2
WT
#3*
WT
WT
300
200
100
c
R60A
WT
#1
#2
#3
WT: 5'...gcacgccggcgcagtctacggaaccgc...3'
#1: 5'...gcacgccggcgcagtctggccaaccgc...3'
#2: 5'...gcacgccggcgcagcagcacggaaccgc...3'
#3: 5'...gcacgccggcgcagtctggccaaccgc...3'
d
Dppa3R60A/R60A
Dppa3R60A/+
Dppa3+/+
bp
300
200
100
e
12 hr
24 hr
48 hr
60 hr
WT
Activated eggs (R60A)
Fertilized embryos (♂R60A x ♀R60A)

Supplementary Figure 3 | Establishment of *Dppa3^{R60A}* mice by CRISPR/Cas9. (**a**) Schematic of the mutagenesis strategy to establish *Dppa3^{R60A}* mice. The sgRNA-targeting sequencing is underlined and the PAM sequence is labeled in bold green. The oligonucleotide used for homologous DNA repair (HDR) is under the target site, with 4 bp changes labeled in red and capitalized. The restriction enzyme (MscI) cleavage site used for genotyping is bold and capitalized. (**b**) Schematic (top) for genotyping *Dppa3^{R60A}* mice from (**a**). Dppa3-F and Dppa3-R are forward and reverse PCR primers, respectively, that amplify a 227 bp fragment that can be cut with MscI in the mutant, but not wild type, allele. PCR amplified tail DNA was digested with MscI and separated on 2% agarose gels (bottom). Correct 182 bp and 95 bp DNA fragments were detected in #1* and #3*, but not #2 mice. (**c**) DNA sequence of #1-3 and wild type (WT) mice. Mutant sequence encoding R60A are enclosed by a red square (left). Samples from #1 and #3 mice were correctly mutated (bold red), but #2 was not (bold green) (right). (**d**) RFLP analysis of offspring from *Dppa3$^{R60A/+}$* matings that results in *Dppa3$^{R60A/R60A}$*, *Dppa3$^{R60A/+}$* and *Dppa3$^{+/+}$* pups. **(e)** Pre-implantation development of fertilized embryos derived from *Dppa3^{WT}* (top) and *Dppa3$^{R60A/R60A}$* (bottom) female mice or activated eggs from *Dppa3$^{R60A/R60A}$* (middle) female mice. Scale bar, 20 μm.

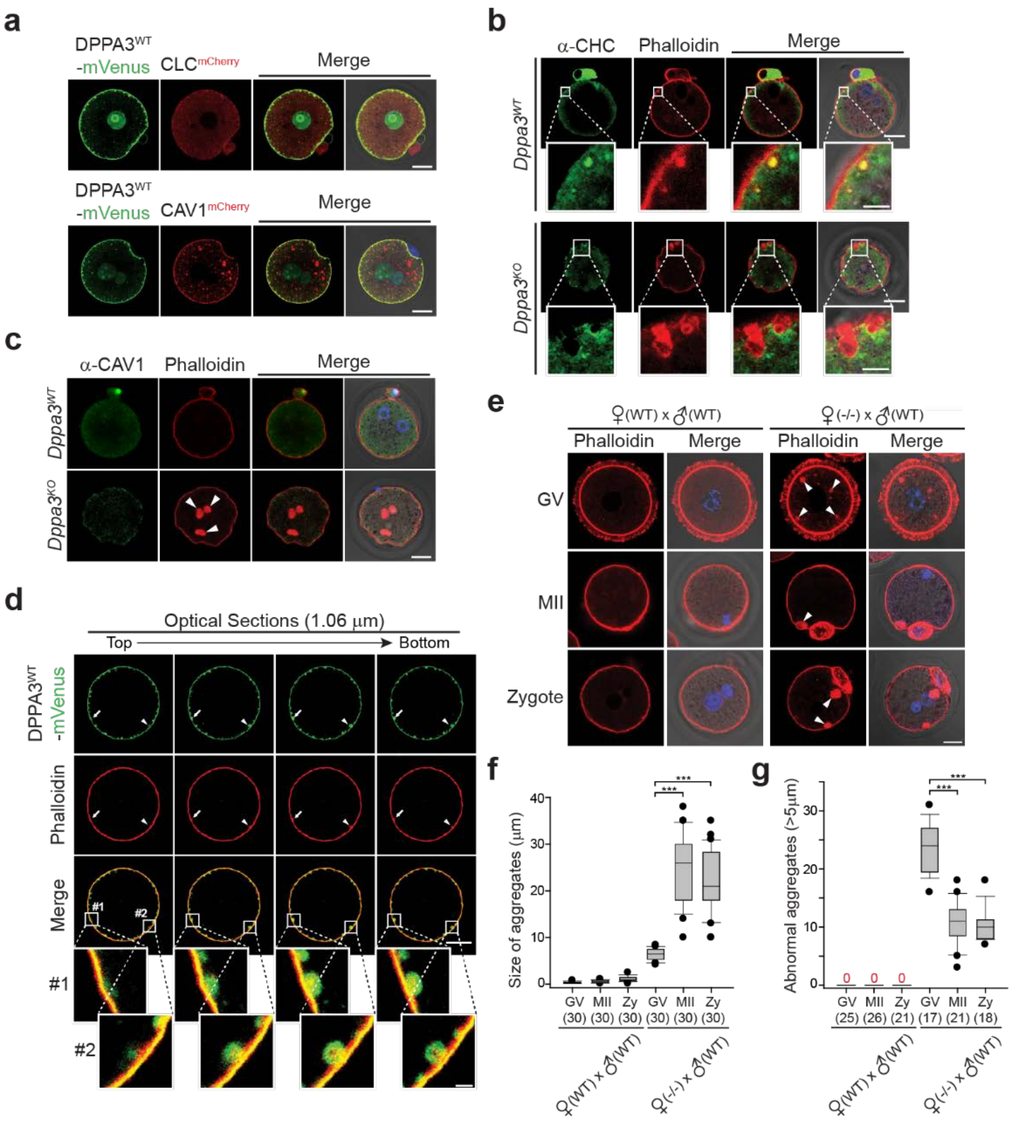

Supplementary Figure 4 | Subcellular localization of clathrin, caveolin1 and aggregates. (**a**) Confocal images of DPPA3^{WT} and clathrin light chain (CLC; top) or caveolin1 (CAV1; bottom) in fertilized 1C zygotes after co-microinjection of *Dppa3^{WT}-mVenus* with *mCherry-CLC* or *mCherry-CAV1*. Images from left to right are: mVenus; mCherry; mVenus and mCherry merged; and mVenus, mCherry, DIC and Hoechst stain merged. Scale bars, 20 μm. (**b**) Confocal images of 1C zygotes from maternal *Dppa3^{WT}* (top) and *Dppa3^{KO}* (bottom) stained with antibodies to clathrin heavy chain (CHC) and with phalloidin. The last two images to the right are merged as in (**a**). Scale bars, 20 μm. Magnified images of squares are on second row of each set. Scale bars, 4 μm. (**c**) Confocal images of 1C zygotes from

maternal *Dppa3*^{WT} (top) and *Dppa3*^{KO} (bottom) as in (**b**), but stained with antibodies to CAV1. Arrowheads, aggregated vesicles. Scale bar, 20 μm. (**d**) Confocal optical sections of DPPA3^{WT}-mVenus (top) stained with phalloidin (middle) and merged (bottom). Scale bar, 20 μm. #1 (arrows) and #2 (arrowheads) squares from merged images are magnified at bottom. Scale bar, 2.5 μm. (**e**) Confocal images of *Dppa3*^{WT} (left) and *Dppa3*^{KO} (right) in GV oocytes, MII eggs and 1C zygotes stained with phalloidin to detect F-actin. Arrowheads, aggregated vesicles. Scale bar, 20 μm. (**f**) Box plots of the size of aggregates (30) in GV oocytes, MII eggs and 1C zygotes from 18-20 different samples. The box includes the mean (horizon line) and data between the 25th and 75th percentile. Error bars indicate the 90th and 10th percentiles and outliers are indicated by dots. *** P <0.001 by two-tailed Student's t-test. (**g**) As in (**f**), but of the number of aggregates.

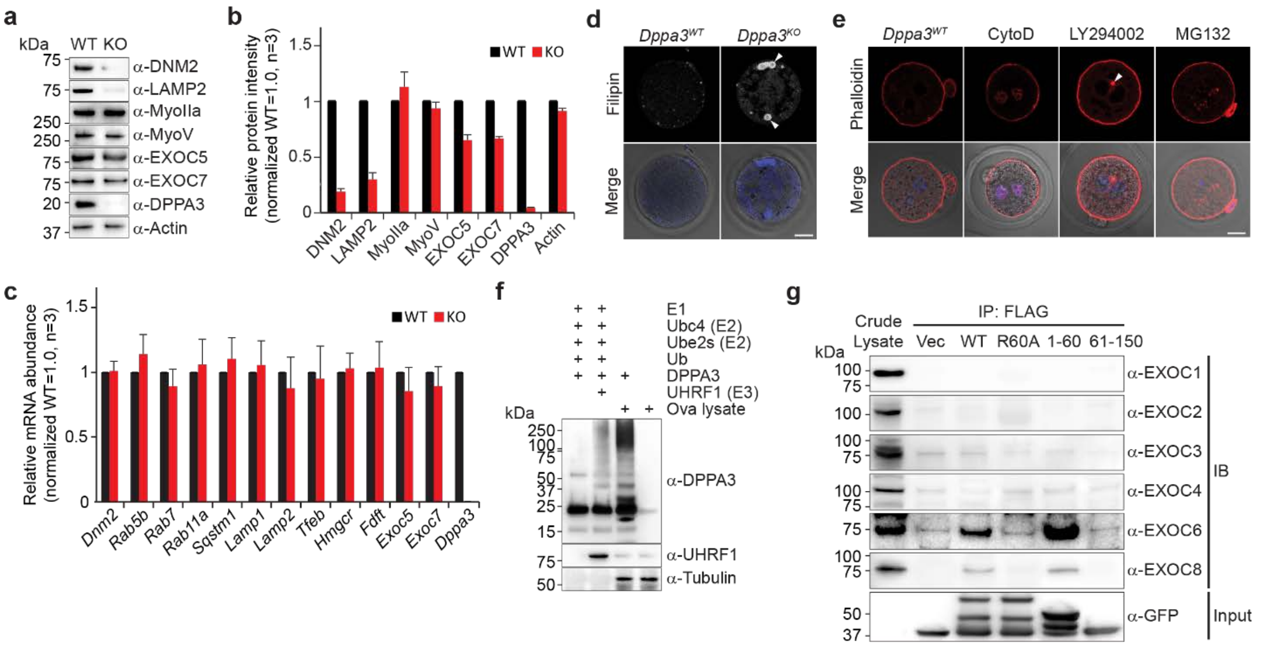

Supplementary Figure 5 | Gene expression, filipin staining and inhibitor assay. (**a**) Immunoblot of ovulated MII egg (30) lysates isolated from *Dppa3*^{WT} and *Dppa3*^{KO} female mice. Antibodies used to probe the blots at left. Myosin IIa and myosin V, abbreviated MyoIIa and MyoV, respectively. (**b**) Quantification of proteins expression in (**a**) in which results of each pair were normalized to WT expression. (**c**) Quantitative RT-PCR of indicated transcripts present in germinal vesicle (GV) oocytes (50) isolated from *Dppa3*^{WT} and *Dppa3*^{KO} female mice. Primer sets are in Table S4 and results of each pair were normalized to WT expression. Error bars, (c,d), SD. (**d**) Confocal images of filipin staining alone (top) and merged with DIC/Hoechst staining (bottom) of ovulated MII eggs from *Dppa3*^{WT} and *Dppa3*^{KO} female mice Arrowheads, aggregated vesicles. Scale bar, 20 µm. (**e**) Confocal images alone (upper) and merged with DIC/Hoechst staining (lower) of *Dppa3*^{WT} 1C zygotes after treatment with indicated inhibitor and staining with phalloidin. Arrowheads, aggregated vesicles. Scale bar, 20 µm. (**f**) Ubiquitin assay with indicated recombinant proteins and ovarian lysates, and immunoblot with indicated antibodies. (**g**) Immunoblot after immunoprecipitation with anti-FLAG antibodies of 293T cell lysates transfected separately with FLAG-tagged *mVenus* (Vec), *Dppa3*^{WT}, *Dppa3*^{R60A}, *Dppa3*$^{1\text{-}60}$, and *Dppa3*$^{61\text{-}150}$. Antibodies used to probe blots on left.

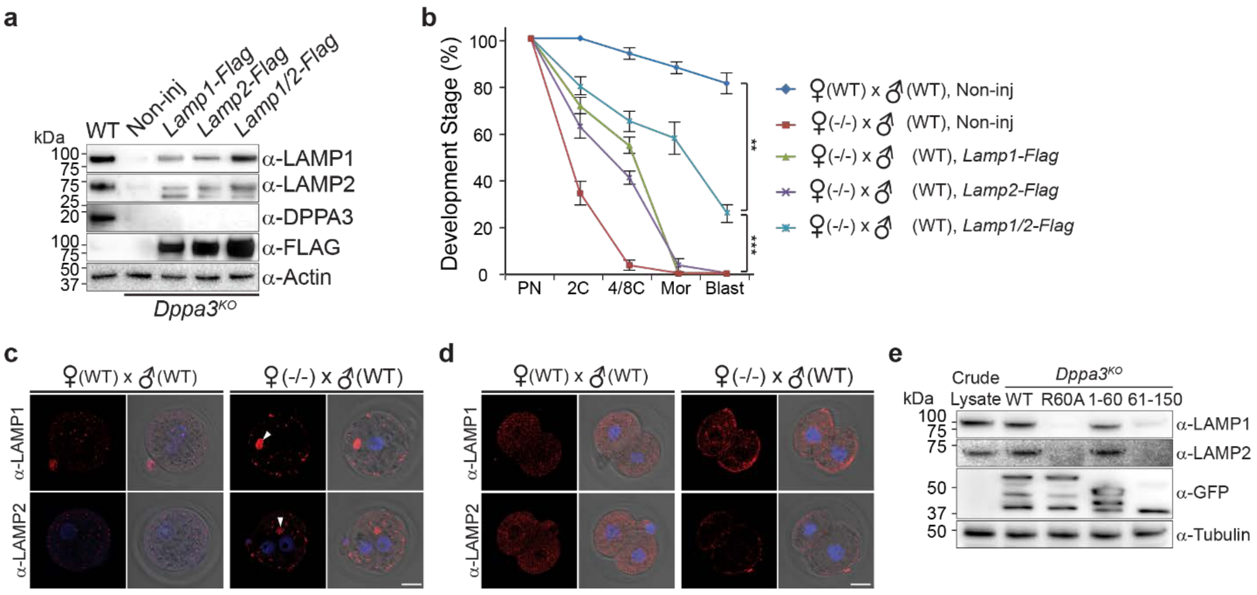

Supplementary Figure 6 | Rescue of arrested pre-implantation embryos by LAMP1/2.

(**a**) Immunoblot with indicated antibodies of 1C zygote lysates from *Dppa3*^{WT} and *Dppa3*^{KO} either un-injected or injected with cRNA encoding LAMP1-FLAG and/or LAMP2-FLAG. Actin was used as a load control. (**b**) Developmental progression of un-injected *Dppa3*^{WT} and *Dppa3*^{KO} 1C zygotes, or *Dppa3*^{KO} 1C zygotes injected with cRNA encoding LAMP1-FLAG, LAMP2-FLAG or both. PN, pronuclei; C, cell; Mor, morula; Blast, blastocyst. Each data point reflects three independent biological samples each with > 20 embryos. Error bars, SD. ** P <0.005; *** P <0.001 by two-tailed Student's t-test. (**c**) Confocal alone and merged with DIC/Hoechst images of 1C zygotes from *Dppa3*^{WT} (left) and *Dppa3*^{KO} (right) injected with cRNA encoding LAMP1/2-FLAG. Zygotes were fixed and stained with anti-LAMP1 and anti-LAMP2 antibodies, respectively. Arrowheads, aggregated lysosomes. Scale bar, 20 μm. (**d**) Same as (**c**), but for 2C embryo. **(e)** Immunoblot with indicated antibodies of 1C zygotes from *Dppa3*^{KO} injected with cRNA encoding WT and truncated DPPA3. Crude lysate from 1C zygotes was used as a positive control. Tubulin served as a load control.

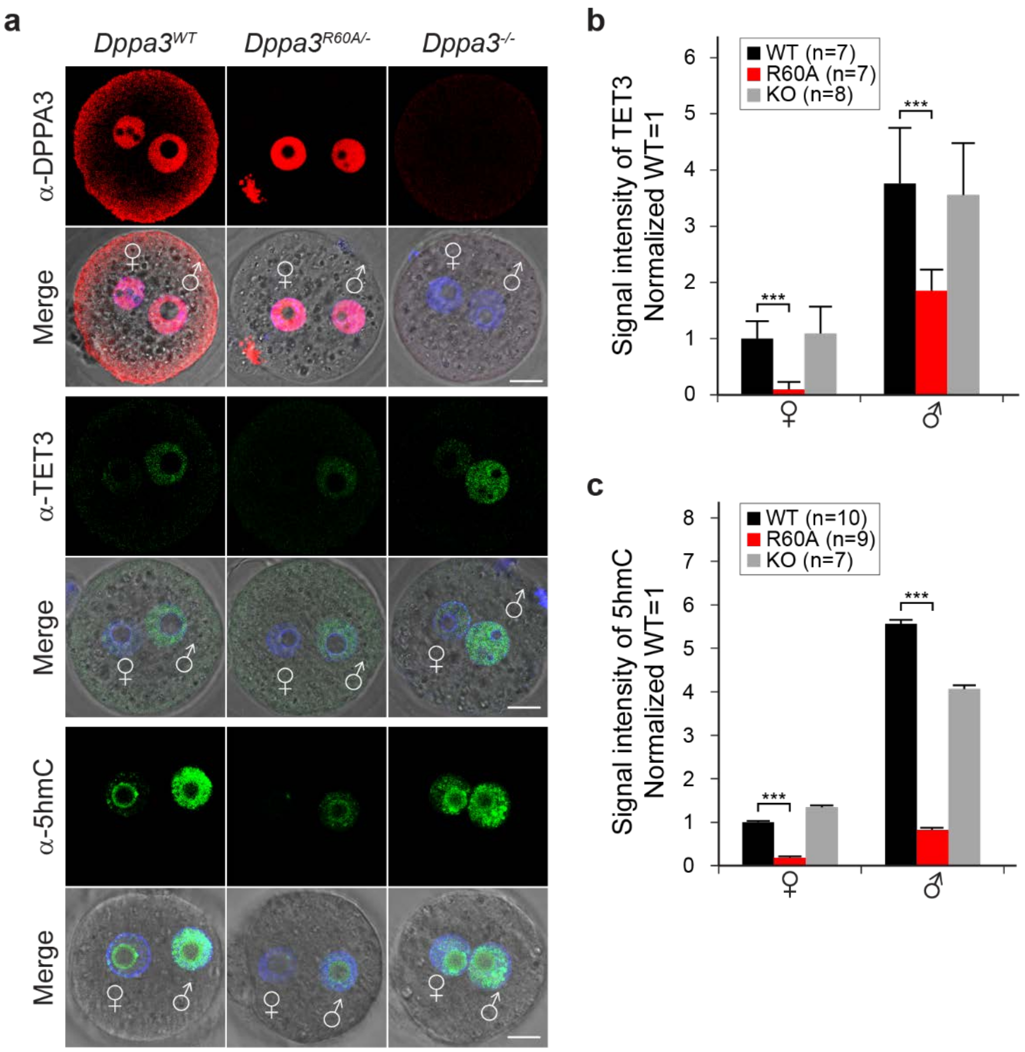

Supplementary Figure 7 | DNA oxidation in *Dppa3*^{WT}, *Dppa3*$^{R60A/-}$ and *Dppa3*$^{-/-}$ 1C zygotes.

(**a**) Confocal images alone and merged with DIC/Hoechst images of DPPA3 (top), TET3 (middle) and 5hmC (bottom) in 1C zygotes from *Dppa3*^{WT}, *Dppa3*$^{R60A/-}$, *Dppa3*$^{-/-}$ females mated with *Dppa3*$^{-/-}$ male mice. *Dppa3*$^{R60A/-}$ mice were obtained from *Dppa3*$^{R60A/R60A}$ females mated with *Dppa3*$^{-/-}$ males. Scale bars, 20 µm. (**b**) Bar graph of relative signal intensity of anti-TET3 antibody in the three genotypes. The Hoechst signals were quantified and normalized to the female pronucleus in *Dppa3*^{WT} after which the intensity of antibody staining was determined. The number of embryos examined is indicated in the legend box. Error bars, SEM. *** P <0.001 by two-tailed Student's t-test. (**c**) Same as (**b**), but for 5hmC.

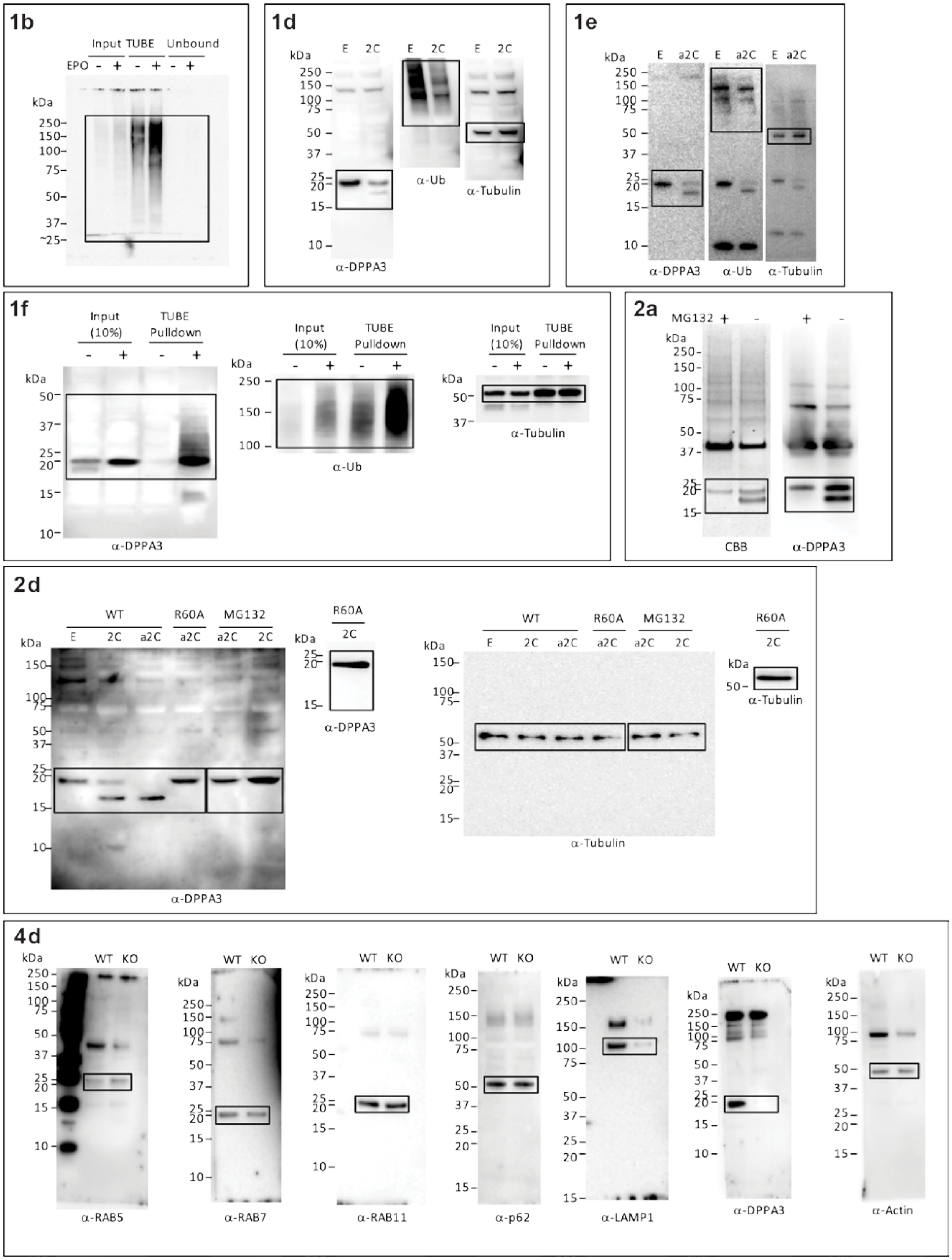

Supplementary Figure 8 | Uncropped images for Figures 1-4.

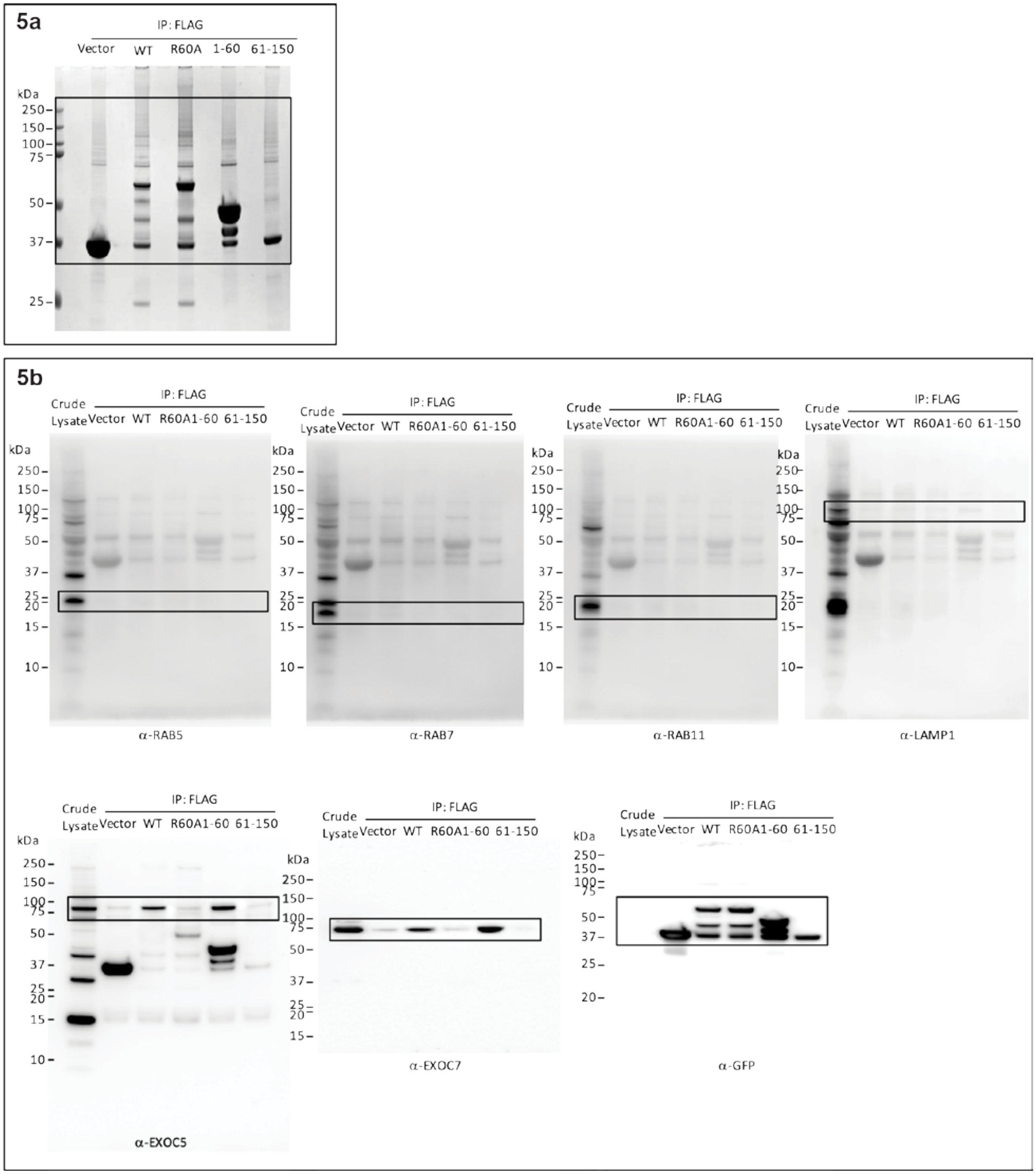

Supplementary Figure 9 | Uncropped images for Figure 5.

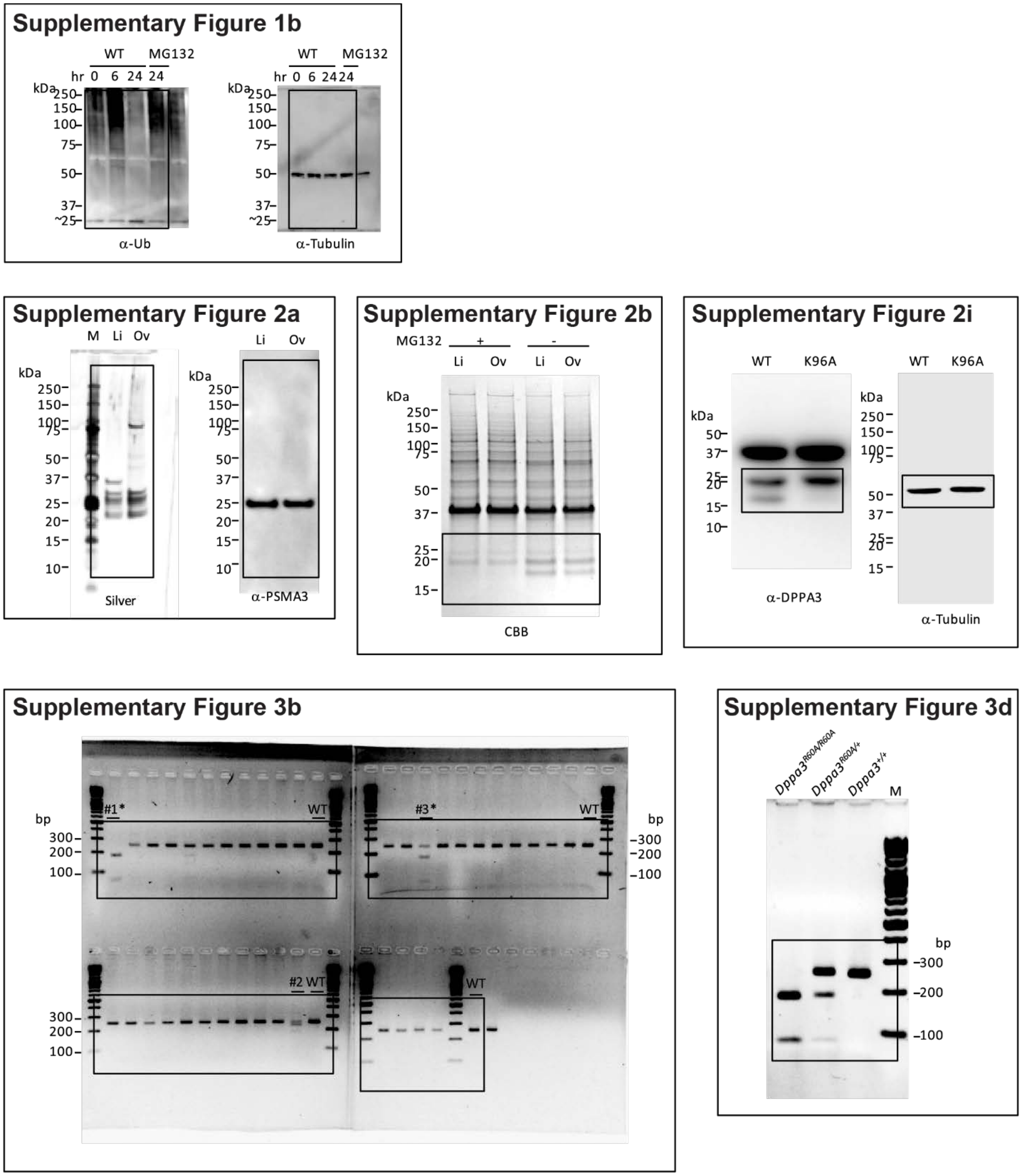

Supplementary Figure 10 | Uncropped images for Supplementary Figures 1-3.

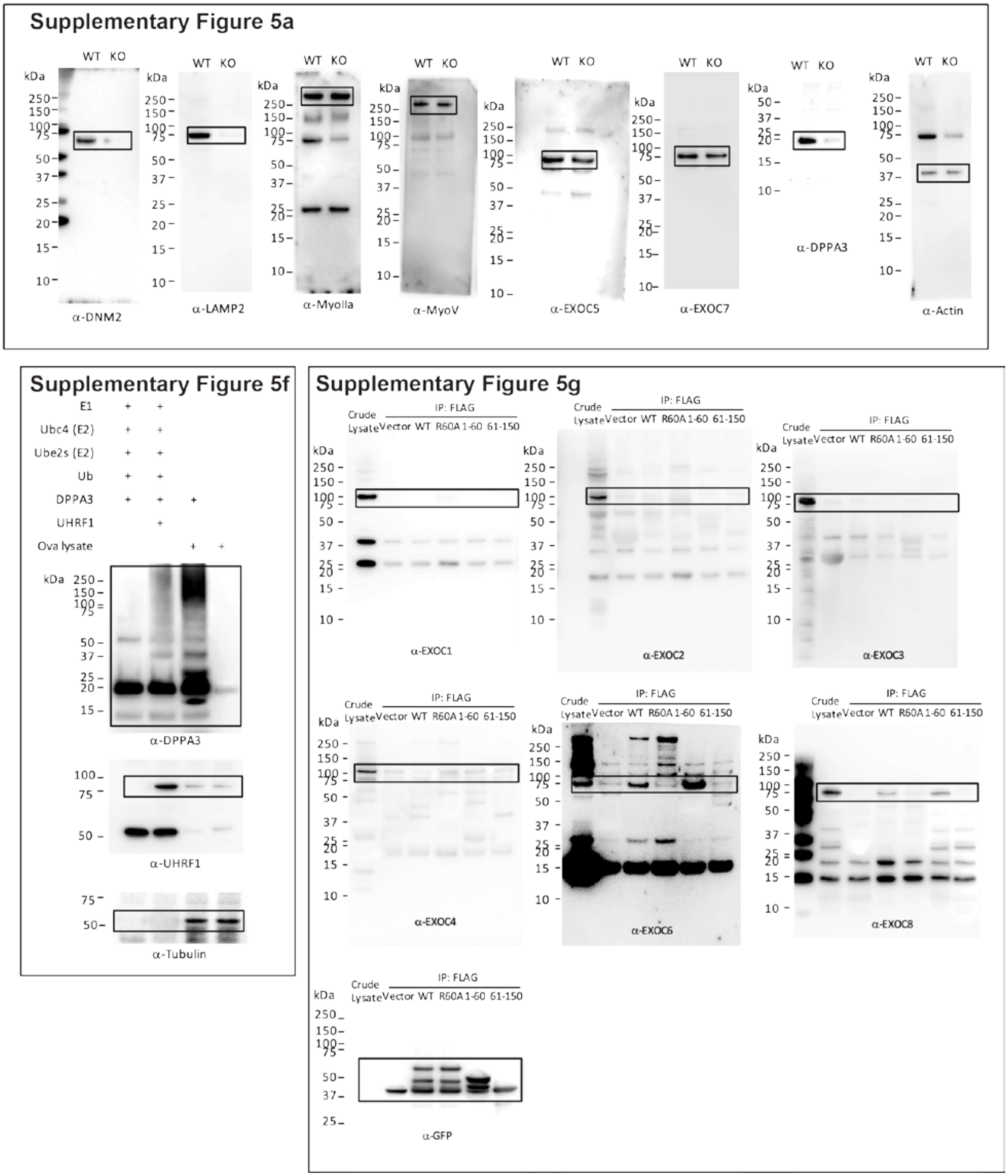

Supplementary Figure 11 | Uncropped images for Supplementary Figure 5.

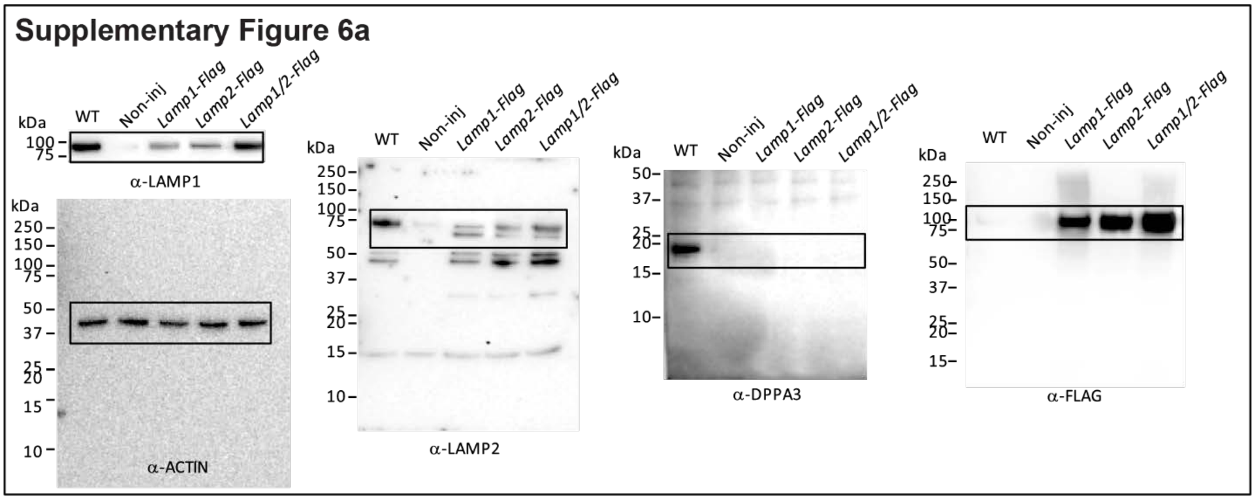

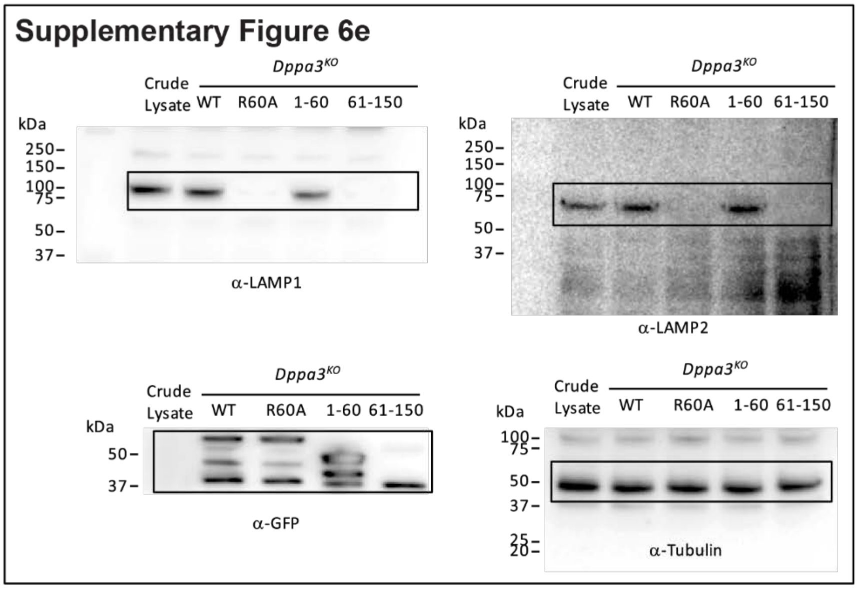

Supplementary Figure 12 | Uncropped images for Supplementary Figure 6.

Supplementary Table 1. Binding partners and peptides of DPPA3

**Protein**	**#**	**Peptides**
EXOC7	1	K.SELIQLVAVTQK.T
	2	K.QTKPEFDQVLQGTAASTK.N
	3	K.YGVEQVGDMIDR.L
	4	K.NMVSILSSFESR.L
	5	K.ETYGAFLQK.F
EXOC5	1	K.QVGDIFSNPETVLAK.L
	2	K.LIQNVFEIK.L
	3	R.SIGTGGIQDLK.E
EXOC8	1	R.QLESGGFEAR.L
UHRF1	1	K.TKVEPYSLTAQQSSLIR.E
	2	K.IERPGEGSPMVDNPMRR.K
	3	K.QMEDGHTLFDYEVR.L
	4	R.GKQMEDGHTLFDYEVR.L
	5	R.NDASEVVLAGER.L
	6	K.VEPYSLTAQQSSLIR.E

Supplementary Table 2. Primers for qPCR

Gene	Sequence (5' -> 3')	Amplicon Size (bp)
Rab5b	CAGGCTGCAATCGTGGTCTAT	285
	ATTCTGGGGTTCGCTCTTTGG	
Rab7	AGGCTTGGTGCTACAGGAAAA	179
	CTTGGCCCGGTCATTCTTGT	
Rab11a	TGGGAAAACAATAAAGGCACAGA	128
	ATGTGAGATGCTTAGCAATGTCA	
Sqstm1	AGGATGGGGACTTGGTTGC	178
	TCACAGATCACATTGGGGTGC	
Lamp1	TGCTCCGGGATGCCACTAT	234
	TGTTGTCCTTTTTCAGGTAGGTG	
Tfeb	CCACCCCAGCCATCAACAC	147
	CAGACAGATACTCCCGAACCTT	
Lamp2	TGTATTTGGCTAATGGCTCAGC	230
	TATGGGCACAAGGAAGTTGTC	
Dppa3	GACCCAATGAAGGACCCTGAA	130
	GCTTGACACCGGGGTTTAG	
Dnm2	TTTGGCGTTCGAGGCCATT	73
	CAGGTCCACGCATTTCAGAC	
Fdft1	ATGGAGTTCGTCAAGTGTCTAGG	202
	CGTGCCGTATGTCCCCATC	
Hmgcr	AGCTTGCCCGAATTGTATGTG	104
	TCTGTTGTGAACCATGTGACTTC	
Exoc5	GACCAGTTGGAAGGGGTAAAC	185
	TCGAAAGGTAACTCCTGGGCA	
Exoc7	CCAGGGACGATCCGTAAGG	92
	AGAGGAATGAGGTTAGAGCCC	
Gapdh	TGAAGCAGGCATCTGAGGG	102
	CGAAGGTGGAAGAGTGGGAG	

* Forward (top) and Reverse (bottom)

Supplementary Table 3. Primary antibodies

**Primary Antibody**	**Company**	**Catalog #**	**Host**	**WB**	**IF**
5hmC	Active Motif	39770	Rabbit		1/500
Actin	SCB	sc-1616	Goat	1/1000	
Caveolin	Abcam	ab2910	Rabbit		1/200
Clathrin heavy chain	Abcam	ab21679	Rabbit		1/200
DPPA3	Abcam	ab19878	Rabbit	1/1000	1/200
Dynamin 2	Abcam	ab3457	Rabbit	1/1000	1/200
EXOC1	Abcam	ab118798	Rabbit	1/1000	
EXOC2	Abcam	ab154688	Rabbit	1/1000	
EXOC3	Abcam	ab156568	Rabbit	1/1000	
EXOC4	Abcam	ab205945	Rabbit	1/1000	
EXOC5	Proteintech	17593-1-AP	Rabbit	1/1000	1/100
EXOC6	Abcam	ab105075	Rabbit	1/1000	
EXOC7	Novus	NBP1-84767	Rabbit	1/100	1/100
EXOC8	Abcam	ab107288	Rabbit	1/1000	
FLAG	WAKO	01822381	Mouse	1/1000	
GFP	SCB	sc-9996	Mouse	1/1000	
His-probe	SCB	sc-8036	Mouse	1/1000	
LAMP1	Abcam	ab24170	Rabbit	1/1000	1/200
LAMP2	Abcam	ab18528	Rabbit	1/1000	1/200
Myosin IIa	Gifted		Rabbit	1/1000	
Myosin V	Gifted		Rabbit	1/1000	
p62	Abcam	ab91526	Rabbit	1/1000	1/1000
PSMA3 (alpha 7)	Gifted		Rabbit	1/1000	
RAB11	Abcam	ab65200	Rabbit	1/1000	1/200
RAB5	Abcam	ab18211	Rabbit	1/1000	1/200
RAB7	Abcam	ab137029	Rabbit	1/1000	1/200
TET3	Active Motif	61743	Rat		1/200
Tubulin (alpha)	SCB	sc-8035	Mouse	1/1000	
Ubiquitin	DAKO	Z 0458	Rabbit	1/1000	

Supplementary Table 4. Secondary antibodies and reagents

**Secondary Antibody**	**Company**	**Catalog #**	**WB**	**IF**	
Donkey anti-Goat IgG-HRP	SCB	sc-2020	1/5000		
Goat anti-Mouse IgG-HRP	SCB	sc-2005	1/5000		
Goat anti-Rabbit IgG-HRP	SCB	sc-2004	1/5000		
Goat anti-Rabbit IgG, Alexa Fluor 555 conjugate	Thermo	A-21428		1/300	
Goat anti-Rabbit IgG, Alexa Fluor 488 conjugate	Thermo	A-11008		1/300	
Goat anti-Rat IgG, Alexa Fluor 488 conjugate	Thermo	A-11006		1/300	
**Reagent**	**Company**	**Catalog #**	**Final conc. or dilution**		
Phalloidin (594)	Invitrogen	A12381	1/100		
Filipin III	Abcam	ab146126	1/100		
LysoTracker-Green	Invitrogen	L7526	5 mM		
pHrodo-Red	Invitrogen	P10361	100 mg/ml		
