# Supplementary material for: Cytoplasmic cleavage of DPPA3 is required for intracellular trafficking and cleavage-stage development in mice
Source: Nat Commun. 2017 Nov 21;8:1643. doi: 10.1038/s41467-017-01387-6 (PMC5696369; doi:10.1038/s41467-017-01387-6)
Supplement: Supplementary file 2 — Description of Additional Supplementary Files [file 41467_2017_1387_MOESM2_ESM.pdf]

Description of Additional Supplementary Files

File Name: Supplementary Data 1
Description: Ubiquitinated Maternal Proteins

File Name: Supplementary Data 2
Description: Mass Spectrometry of 20S Proteasome
